# Diversified Shifts in the Cross Talk between Members of the Gut Microbiota and Development of Coronary Artery Diseases

**DOI:** 10.1128/spectrum.02804-22

**Published:** 2022-10-27

**Authors:** Tao Zhang, Haiqing Ren, Zhihui Du, Tong Zou, Xuefeng Guang, Yanan Zhang, Yuqing Tian, Lei Zhu, Jiangkun Yu, Xue Yu, Zhigang Zhang, Hailong Dai

**Affiliations:** a State Key Laboratory for Conservation and Utilization of Bio-Resources in Yunnan, School of Life Sciences, Yunnan University, Kunming, Yunnan, People’s Republic of China; b Department of Cardiology, Key Laboratory of Cardiovascular Disease of Yunnan Province, Yan’an Affiliated Hospital of Kunming Medical University, Kunming, People’s Republic of China; c Department of Cardiology, Beijing Hospital, National Center of Gerontology, Institute of Geriatric Medicine, Chinese Academy of Medical Sciences, Beijing, People’s Republic of China; d Department of Critical Care Medicine, The Affiliated Hospital of Qingdao University, Qingdao, Shandong, People’s Republic of China; e Department of Cardiology, Affiliated Hospital of Panzhihua University, Panzhihua, People’s Republic of China; f Department of Ultrasonography, Ordos Central Hospital, Ordos, Inner Mongolia, People’s Republic of China; Huazhong University of Science and Technology; State Key Laboratory for Bioactive Substances and Functions of Natural Medicines, Beijing Key Laboratory of New Drug Mechanisms and Pharmacological Evaluation Study, Institute of Materia Medica, Chinese Academy of Medical Sciences and Peking Union Medical College; Federal Research and Clinical Centre of Physical and Chemical Medicine

**Keywords:** CAD, gut microbiota, mild coronary stenosis, stable angina, unstable angina, acute myocardial infarction, random forest, PCA, clinical microbiome

## Abstract

Coronary artery disease (CAD) is one of leading causes of mortality worldwide. Studies on roles that the gut microbiota plays in development of atherosclerosis or acute myocardial infarction (AMI) have been widely reported. However, the gut microbiota is affected by many factors, including age, body mass index (BMI), and hypertension, that lead to high CAD risk. However, the associations between gut microbiota and CAD development or other CAD risk factors remain unexplored. Here, we performed a 16S RNA gene sequencing analysis of 306 fecal samples collected from patients with mild coronary stenosis (MCS; *n* = 36), stable angina (SA; *n* = 91), unstable angina (UA; *n* = 48), and acute myocardial infarction (AMI; *n* = 66) and 65 non-CAD controls. Using a noise-corrected method based on principal-component analysis (PCA) and the random forest algorithm, we identified the interference with gut microbial profiling of multiple factors (including age, gender, BMI, and hypertension) that potentially contributed significantly to the development of CAD. After correction of noise interference from certain interfering factors, we found consistent indicator microbiota organisms (such as *Vampirovibrio*, *Ruminococcus*, and *Eisenbergiella*) associated with the presence of MCS, SA, and AMI. Establishment of a diagnostic model revealed better performance in early CAD than clinical indexes with indicator microbes. Furthermore, indicator microbes can improve the accuracy of clinical indexes for the diagnosis of AMI. Additionally, we found that the microbial indicators of AMI *Sporobacter* and *Eisenbergiella* showed consistent positive and negative correlations to the clinical indexes creatine kinase (CK) and hemoglobin (Hb), respectively. As a control indicator of AMI, *Dorea* was negatively correlated with CK but positively correlated with Hb.

**IMPORTANCE** Our study discovered the effect of confounding factors on gut microbial variations and identified gut microbial indicators possibly associated with the CAD development after noise correction. Our discovered indicator microbes may have potential for diagnosis or therapy of cardiovascular disorders.

## INTRODUCTION

Cardiovascular disease (CAD) is one of the most common cardiovascular diseases in the world and has been a leading cause of death in developing and developed countries in recent years (1). CAD develops as atherosclerosis formed in coronary arteries, an inflammation process associated with higher blood cholesterol levels. Several factors, such as lifestyle, genetics, and environmental changes, influence the development of CAD (2, 3). In addition, smoking, diabetes, obesity, hyperlipidemia, and hypertension also serve as risk factors for the occurrence of CAD (4–8). Death rates from CAD have declined due to popular therapeutic interventions and techniques such as angioplasty and stent placement, as well as drugs such as aspirin, statins, and β-blockers, yet the burden of disease remains high (9, 10). Therefore, new interventions still need to be explored.

Recently, increasing studies have reported associations between gut microbes and CAD development (11). Cholesterol metabolism and bile acid production, both of which are significantly regulated by gut microbes, modulate systemic cholesterol levels (12) and affect the development of CAD in direct or indirect ways (13). For healthy individuals, some gut symbiotics, such as Faecalibacterium prausnitzii, *Roseburia*, and Alistipes putredinis, produce short-chain fatty acids (SCFAs) (14), including acetate, propionate, and butyrate, that can block the synthesis of cholesterol and/or redirect it to the liver to maintain a lower blood cholesterol level. Some others, such as *Lactobacillus*, *Eubacterium*, and *Bacteroides* (15–17), promote the conversion of gut cholesterol to unabsorbable coprostanol and fecal excretion to protect hosts from higher levels of systemic cholesterol (18). Once in dysbiosis, gut microbes promote the development of CAD. The gut bacteria were reported to accelerate the conversion of choline to trimethylamine-*N*-oxide (TMAO), and TMAO elevation significantly associated with higher risk of myocardial infarction or stroke and even death (19). Besides direct involvement, altered gut microbiota can also raise inflammation levels and promote the formation and development of atherosclerosis in indirect ways by interacting with the host immune system (20, 21). However, although the use of pre- and probiotics for the control of CAD is increasing, their effectiveness remains at alarmingly low levels (22, 23).

Many factors, such as age, gender, race, excess weight, diabetes, and hypertension, significantly contribute to CAD occurrence and development, and many of these have been widely reported to affect the composition of the gut microbiota (24). The interference of the gut microbiota with risk factors hampered the precision interpretation of the role of gut microbes in CAD patients. Many studies solved the problem by controlling the inclusion criteria with similar ranges for matched interfering factors (25–27). However, the conclusions may still change when different ranges or factors are taken into consideration for different studies, especially studies of therapy. Some studies clustered microbial samples into various community types using an unsupervised approach for correction of interindividual variance (28). The specific community type sharing similar composition represented an objective final state shaped by multiple interfering factors, but it was inconclusive whether the interference from a certain interfering factor in a specific community type contributed to disease development or background noise. The effects of a given interfering factor may be different with regard to its contribution to the formation of different community types. Controlling the noise influence of potential factors on gut microbiota composition empirically may lead to confusing results. While the method is effective in removing background noise in gut microbiota data for disease studies, Briscoe and colleagues (29) discussed current widely used methods and the performance of methods which offered hints for our noise correction methods.

To identify gut microbes associated with the development of CAD, we collected fresh fecal samples from CAD patients with mild coronary stenosis (MCS), stable angina (SA), unstable angina (UA), and acute myocardial infarction (AMI) and non-CAD controls. To control the interference of multiple non-CAD factors or noise (such as age, gender, body mass index [BMI], and hypertension) with the gut microbiota, we quantified the effect of non-CAD factors on gut microbiota in CAD patients by applying a principal-component analysis (PCA) and a random forest algorithm-based method. Our noise-corrected results may provide new insights into the potential role of the gut microbiota in CAD development, diagnosis, and therapies.

## RESULTS

### Sample collection and preprocessing of 16S RNA sequencing data.

Following diagnosis guideline of coronary syndromes (30), 241 fecal samples from 36 MCS patients, 91 SA patients, 48 UA patients, and 66 AMI patients were collected as disease samples, and samples from 65 non-CAD patients were collected as controls. Clinical information for the 306 subjects studied is presented in Table S1 in the supplemental material.

After quality control (see Materials and Methods), a total of 7,038,360 high-quality sequences were obtained, with an average of 22,852 ± 3,961 sequences per sample. With a 97% sequence identity threshold, a total of 3,644 operational taxonomic units (OTUs) with 167 ± 64 OTUs per sample were finally obtained. To eliminate the bias resulting from different sequencing depths across samples, the OTU abundance table was subsampled at an even depth of 10,000 reads without replacement where the richness almost reached a plateau according to the rarefaction curve (see Fig. S1 and Table S2).

### Interference with gut microbiota by age, BMI, hypertension, and gender in different CADs with different degrees of interference.

After taxonomy classification, abundance at the genus level was applied for identification and correction of confounding factors with our PCA and random forest-based method (Fig. S2).

**FIG 1:**
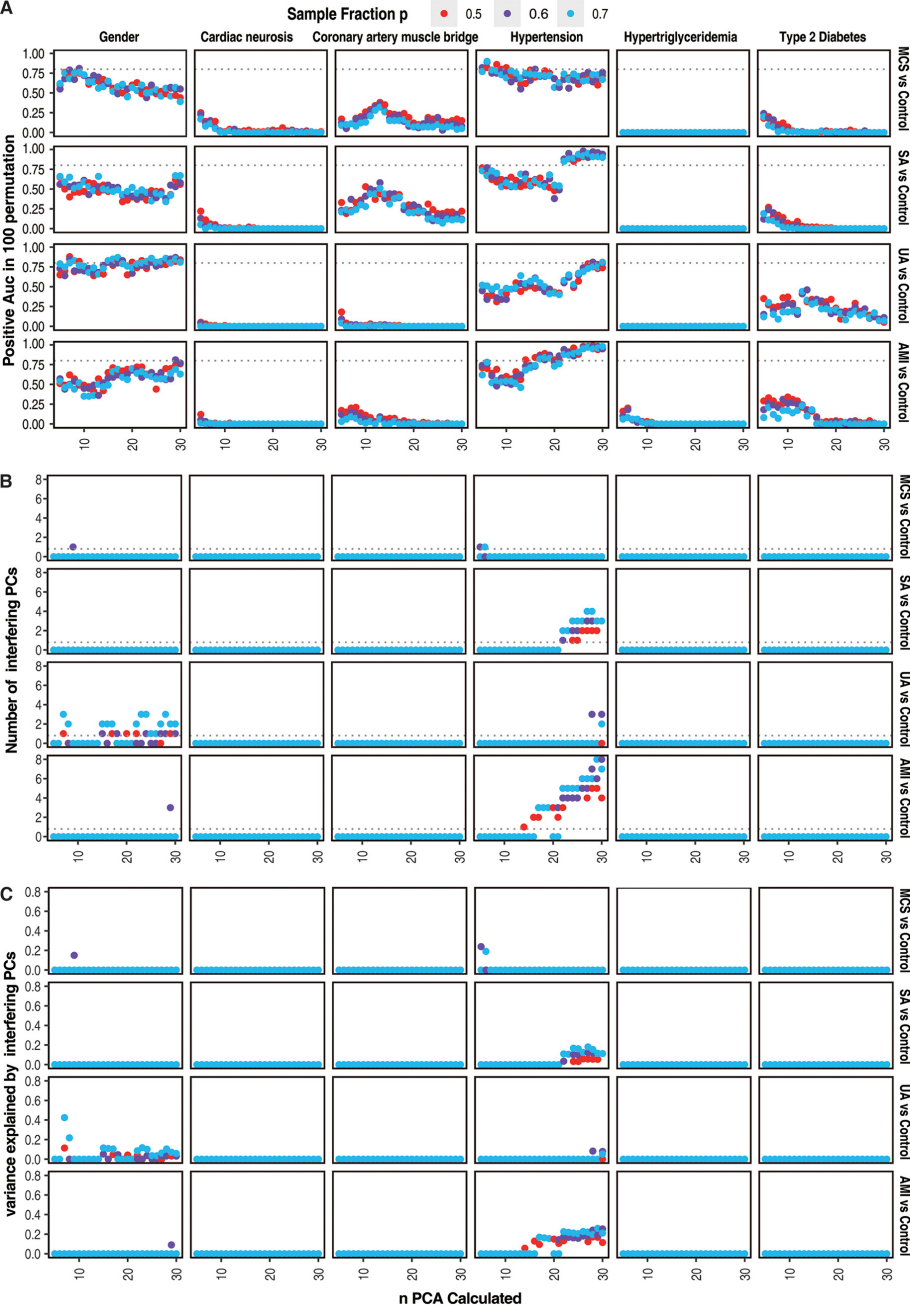
Details in identification of discrete interfering factors in MSC versus controls, SA versus controls, UA versus controls, and AMI versus controls. (A) Identification of interfering factors according to AUC values from discrete confounding factors. Points in plots represent the number of testing runs with AUC values of >0.5 in 100 permutations by the random forest model, which was trained with PCA scores calculated using *n* PCs (*n* ranges from 5 to 30). In each training run, *p* samples with certain confounding factor combined with *p* samples without the certain confounding factor were sampled and merged as a training set, and the rest (1 − *p*) of all samples served as testing samples. *p* ranges among 0.5, 0.6, and 0.7 (points in red, purple, and blue, respectively). A positive AUC rate of 0.8 (80 testing runs with AUC values of >0.5 in 100 permutations) is indicated with the dashed line. Only confounding factors with more than 1 point showing a positive AUC rate of ≥0.8 with any value of *n* (number of calculated PCs) or *p* (sampled samples) were identified as interfering factors. (B) Number of interfering PCs contributing to the discrete interfering factors. In training and testing of random forest models for classification of certain interfering factors (positive AUC rate ≥ 0.8) with PCA scores in 100 permutations, PCs with an MDA of >0 in more than 80 runs were identified as interfering factors. Points represent the numbers of interfering PCs with any value of *n* calculated PCs or *p* samples tested. *p* ranges among 0.5, 0.6, and 0.7 (points in red, purple, and blue, respectively). The value of 1 is marked with a dashed line. (C) Variance explained by interfering PCs for each discrete interfering factor. Points represent variance, which was calculated as sum(score of interfering PCs of *n* calculated PCs)/sum(score of all *n* PCs) for each interfering factor.

For discrete confounding factors, due to a least common appearance in more than 10 of 306 samples for estimation of the area under the curve (AUC), 5 of 27 confounding diseases (type 2 diabetes, hypertriglyceridemia, hypertension, coronary artery muscle bridge, and cardiac neurosis) combined with gender were selected as candidates for downstream identification (Table S2). After training and testing with PC scores (number of PCs calculated [*n*] and fraction of samples tested [*p*]) with 100 replicates, the random forest classifier successfully differentiated gender as well as hypertension in MCS cases versus controls, UA cases versus controls, and AMI cases versus controls, with an AUC of >0.5 more than 80 times in at least 1 *n*-*p* parameter combination. This suggested gut microbiota interference of both gender and hypertension in these groups (Fig. 1A; Table S3). In the comparison of SA cases versus controls, only hypertension was successfully identified (Fig. 1A; Table S3). As only PCs calculated at the *n*-*p* parameter combinations where interfering factors were successfully identified and with a mean decrease accuracy (MDA) of >0 more than 80 times in 100 permutations in the training set were identified as interfering PCs, this showed that the number of PCs contributing to interfering factors varied in different disease groups and that as calculated PCs increase, the number of interfering PCs also tends to increase (Fig. 1B; Table S4). As more PCs were calculated, the variance accounted for by each PC became more specific, and this tendency indicated a better performance with more PCs calculated. However, when the associated variance by interfering PCs for a given interfering factor was summed, the total variance explained by that interfering factor likely kept even (0.2 to 0.3) as the number of calculated PCs increased in each disease group (Fig. 1C; Table S5).

For discrete confounding factors that did not reach our identification threshold, this suggested that the confounding factors did not have high enough confidence for the lower replication rates in 100 permutations, rather than a lack of interference in gut microbiota composition. For example, coronary artery muscle bridge and type 2 diabetes also showed a moderately positive AUC rate in 100 permutations; however, their positive AUC rates (around 0.5 for the highest AUC rate) were much lower than those of gender and hypertension. For a reliable identification and correction, coronary artery muscle bridge and type 2 diabetes were not taken into consideration in downstream correction due to the probably low recurrence rate.

**FIG 2:**
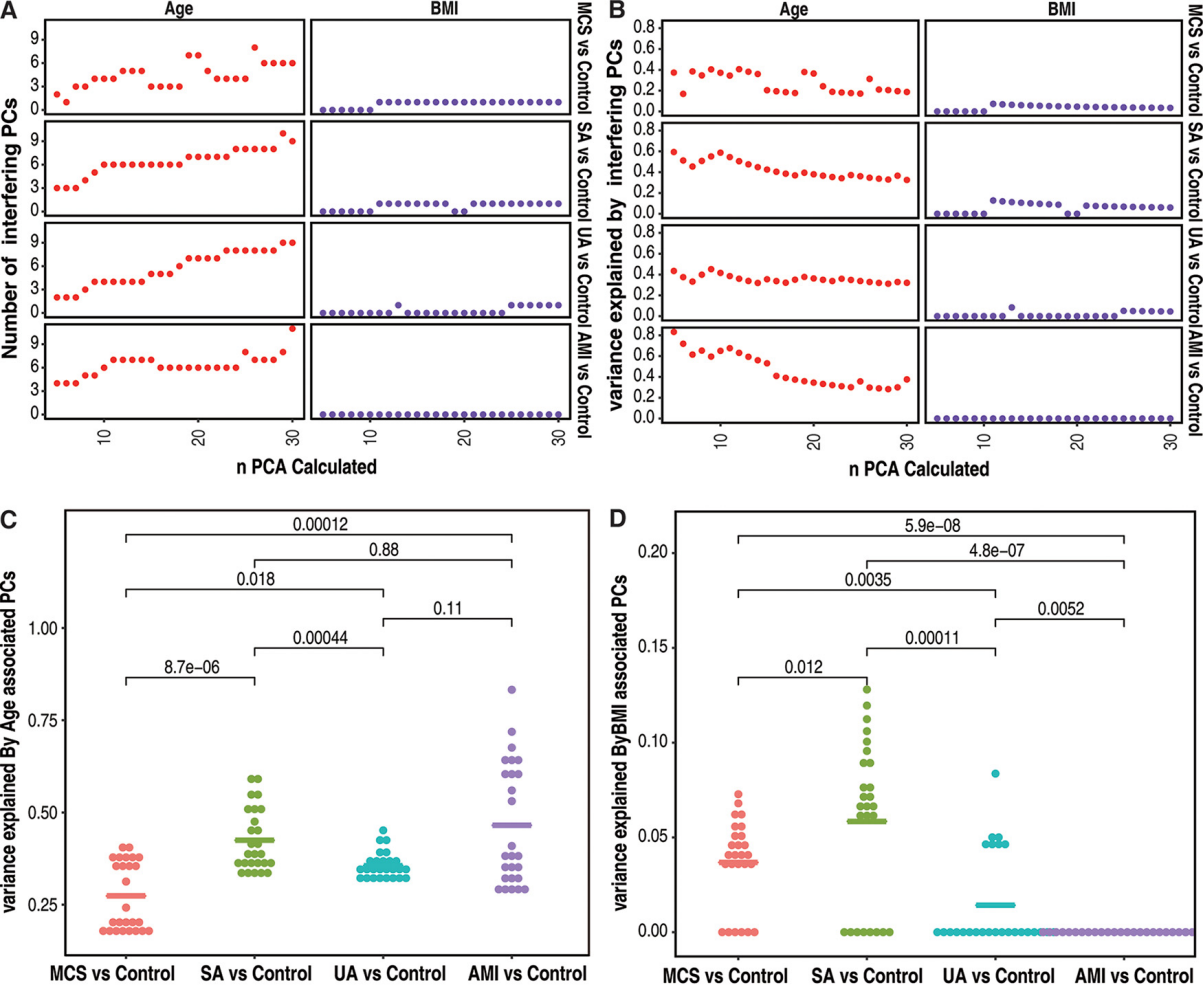
Details of the identification of continuous interfering factors in MSC versus controls, SA versus controls, UA versus controls, and AMI versus controls. (A) Number of interfering PCs contributing to age- and BMI-associated interference. Points represent the number of interfering PCs associated with age and BMI with any *n* calculated PCs according to *P* value in the GLM (Poisson distribution). PCs with *P* values of <0.05 were considered interfering PCs. (B) Variance explained by interfering PCs for age and BMI. Points represent variance, which was calculated as sum(score of interfering PCs of *n* calculated PCs)/sum(score of all *n* PCs) for age or BMI. (C) Comparison of age interference with variance explained by age-associated interfering PCs among groups. A *P* value of <0.05 was considered significant (Wilcoxon test). (D) Comparison of BMI interference with variance explained by BMI-associated interfering PCs among groups. A *P* value of <0.05 was considered significant (Wilcoxon test).

For continuous confounding factors, such as age and BMI, significantly associated PCs were found according to *P* value in the generalized linear mixed model (GLM) in different disease groups. For age, it was found that when more PCs were calculated, more associated interfering PCs were found (Fig. 2A; Table S6), but for BMI, the interfering PC number seemed to be 1 no matter how many PCs were calculated (Fig. 2A; Table S6). Unlike that found in discrete factors, though more PCs were calculated, the total variance explained by age tended to decrease before reaching a plateau in each group, and the tendency became more obvious in the comparison of AMI versus the control group (Fig. 2B; Table S7). For BMI, variance likely showed same tendency as age, though with much lower fluctuations (Fig. 2B; Table S7). Though age and BMI showed overall interference in all 4 groups, the degree of influence varied among groups. Among groups, age showed the significantly lowest influence in MCS versus controls and the significantly highest variance in UA versus controls and AMI versus controls (Fig. 2C). BMI showed the significantly highest interference in SA versus controls and no significant interference in AMI versus controls (Fig. 2D). The failure to identify BMI-associated PCs did not rule out the possibility of BMI interference in AMI, which might be too slight to be identified for significance. Differences in explained variance for different or even the same interfering factors in different groups suggested the variation in the degree of influence by interfering factors.

### Noise- or disease-related roles of interfering confounding factors in different CAD groups.

With the *n*-*p* parameter combination used for interference factor identification, associated PCs of discrete and continuous confounding factors were corrected as described in Materials and Methods and with corrected abundance, the random forest model for case/control classification was trained and tested to test the correction performance among different *n*-*p* combinations. AUC was calculated before and after correction with all, individual, combined, or no interfering-factor-associated PCs in 100 permutations. AUC changes before and after correction for the same parameter combination were used to estimate the role of interfering factors. AUC changes after correcting certain interfering factor differed for different *n*-*p* parameter combinations due to randomly chosen samples; however, it was speculated that if the correction of a given interfering factor resulted in significant AUC elevation despite changes of *n*-*p* combinations, the interfering factor was likely to be noise associated; otherwise, if the AUC dropped significantly after correction of a given interfering factor across all *n*-*p* combinations, the interfering factor probably contributed to the case/control difference and played a role in disease.

**FIG 3:**
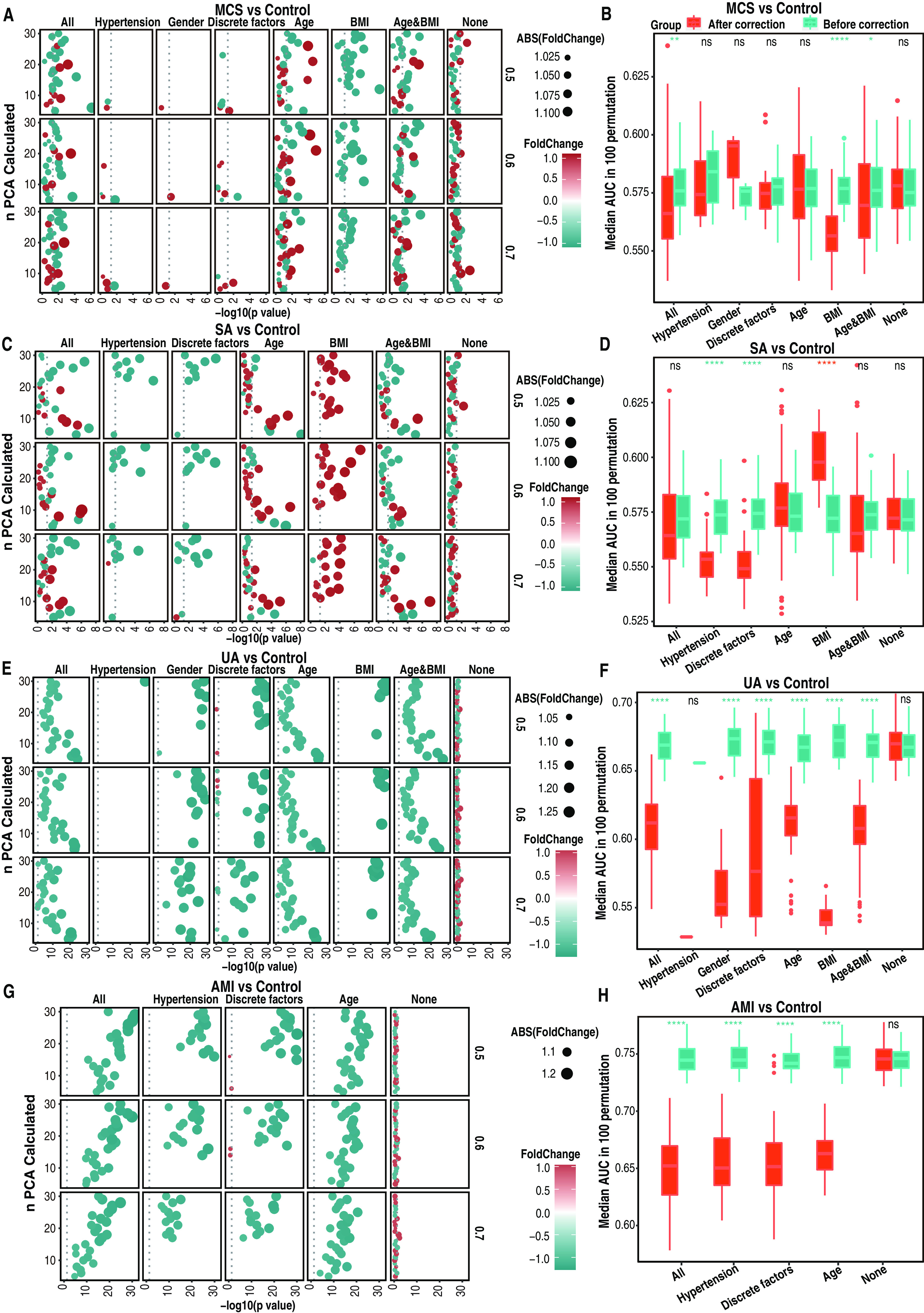
Details of the changes of AUC after interfering factor correction in classification of case/control samples in the random forest model. Specific AUC changes in classifying case/control samples in the random forest model for specific *n* PCs and *p* sampled samples as well as overall median AUC changes across all *n* PCs after correction of the same interfering factors were analyzed in MSC versus controls (A and B), SA versus controls (C and D), UA versus controls (E and F), and AMI versus controls (G and H). Points in each left plot represent the specific AUC changes in 100-permutation classifications of case/control samples after correction of a given interfering factor or interfering factor combination for specific *n* calculated PCs (*n* ranges from 5 to 30) and *p* samples (*p* ranges among 0.5, 0.6, and 0.7). If median AUC after correction was greater than or equal to median AUC before correction, the fold change was calculated as (median AUC after correction/median AUC before correction); otherwise, it was −(median AUC before correction/median AUC after correction). *P* values of AUC changes were analyzed using all AUCs after and before correction of specific interfering factors in 100 permutations with the Wilcoxon test. Color of points changes as fold change changes. Size of points changes according to the absolute value of fold change. The box plot in each right plot represents the overall AUC change, which was analyzed with the median AUC of 100 permutations across all *n* PCs calculated and *p* samples tested, comparing results before and after correction using the Wilcoxon test. *, 0.01 < *P* < 0.05; **, 0.001 < *P* < 0.01; ***, 0.0001 < *P* < 0.001; ****, *P* < 0.0001. The factor “all” means that AUC changes resulted from correcting with all interfering factors together; “discrete factors” indicates correction with discrete factors together; “Age&BMI” indicates correction with age and BMI together; “none” indicates that correction was done with no interfering factors (i.e., the 100 permutation classifications were done again with the same *n*-*p* parameter combinations as before correction).

In the comparison of MCS versus controls, after hypertension, gender, age, and BMI, as well as in the negative control (no interfering-factor correction) were corrected, different AUC changes were found (Table S8). PCs associated with gender and hypertension were identified in only a few *n*-*p* parameter combinations, and only 1 significant increase in AUC was found regardless of whether correction was made alone or in combination (Fig. 3A). Though little interference was found, as correction of gender and hypertension in combination and separately was performed with randomly tested samples, similar results for correcting together and alone also suggested the robustness of our methods (Fig. 3A). Although correction with age alone showed significant AUC changes with different *n*-*p* combinations (Fig. 3A), the overall median AUC showed no significant changes (Fig. 3B). Correction with BMI alone showed a significantly consistent drop in AUC at each *n*-*p* parameter combination (Fig. 3A) and significant decreases in median AUC across all parameter combinations (Fig. 3B), implying the likely disease-associated role of BMI in MCS versus controls. This finding was further confirmed through correction with age combined with BMI or alone. When age alone was corrected, the overall median AUC across all parameter combinations changed without significance, while upon correction of age and BMI in combination, the median AUC showed a significant drop (Fig. 3B). Upon correction for every factor alone except BMI, median AUCs across all parameters showed no significance (Fig. 3A). However, a significant median AUC drop was found after correction with the combination of all factors (Fig. 3B). In total, these results indicated that the interference of BMI in the MCS versus the control group likely contributed to disease development (Fig. 3A and B).

In the comparison of SA versus controls, hypertension, age, and BMI were identified as interfering factors due to significant changes in AUC values during correction for each interfering factor with different *n*-*p* combinations (Fig. 3C). Correction of hypertension brought about consistently significant overall drops for each parameter combination (Fig. 3C) and a significant median AUC drop across all *n*-*p* parameter combinations (Fig. 3D), indicating a potential disease-associated role for hypertension in SA versus controls. With respect to age, no significant changes in median AUC across all *n*-*p* combinations after correction were found (Fig. 3D), which indicates that age was likely not a disease-associated interfering factor, but a significant elevation of AUC was also found with many parameter combinations (Fig. 3C), suggesting an AUC elevation potential for correction. Unlike in the comparison of MCS versus controls, correction of BMI in SA versus controls brought about significant AUC elevation at all *n*-*p* parameter combinations (Fig. 3C) as well as significant increases in median AUC (Fig. 3D), and furthermore, median AUC after BMI correction was the highest among those corrected for every other interfering factor (Fig. 3D), indicating a noise-associated role for BMI in SA versus controls. When age and BMI were corrected in combination, diverse AUC changes were also seen, but no significance was found in median AUC changes at matched parameter combinations (Fig. 3C and D); correction of age and BMI in combination might lead to a neutralization in the AUC elevation that resulted from correcting BMI alone (Fig. 3D).

In the comparison of UA versus controls, hypertension, gender, age, and BMI were identified as interfering factors, and in the comparison of AMI versus controls, only hypertension and age were interfering factors. Regardless of correction with each factor alone or in combination in both groups, the AUC for each parameter combination (Fig. 3E and G) and the median AUC across all parameter combinations (Fig. 3F and H) were all significantly decreased compared to those before correction, suggesting disease-associated roles for hypertension, gender, age, and BMI in UA versus controls and possible disease-associated roles for hypertension and age in AMI versus controls.

### Identification and correction of noise-associated interfering factors.

The *n*-*p* parameter combination with the highest AUC elevation after correction of highly putative noise interfering factors was selected for the identification of microbial indicators associated with CADs. In MCS versus controls (Fig. 3), age correction with the parameter combination where *n* is 26 and *p* is 0.6, leading to the maximal AUC elevation (median AUC change, 1.1024-fold), was selected (Fig. S3A), as age correction also brought about significant AUC elevation at certain *n*-*p* parameter combinations despite the absence of a significant median AUC drop across all *n*-*p* combinations. To estimate the correction performance, nonmetric multidimensional scaling (NMDS) and analysis of similarity (ANOSIM) were performed based on the corrected genus centered log ratio (CLR) matrix and showed a better separation of MCS and control samples with a much lower *P* value (0.003), a greater stress value (0.289), a greater *R* value (0.0797) compared with the *P* value (0.047), stress value (0.286), and *R* value (0.0509) obtained before correction (Fig. S3B), suggesting the effectiveness of correction.

As in the comparison of SA versus controls, BMI was clearly identified as a noise-associated interfering factor (Fig. 3). Though there was still a significant AUC elevation in the correction of age and BMI in combination for some parameter combinations, these combinations did not raise BMI-associated PCs (Fig. 3C). Thus, it was speculated that correcting BMI alone would be more useful in the comparison of SA versus controls. With the parameter combination of an *n* value of 25 and a *p* value of 0.6, which led to the greatest AUC elevation in BMI correction (Fig. S3C), a corrected CLR matrix was applied in NMDS and ANOSIM. Though there was no significant separation between SA and control samples either after or before correction, there was still a *P* value drop from 0.525 to 0.383 and *R* value elevation from −0.0011 to 0.0037 after correction (Fig. S3D). In ANOSIM, a negative *R* value usually suggests no difference between inter- and intragroup analysis, while an *R* value of >0 usually suggests that differences exist; thus, it also likely suggests effective correction.

In the comparisons of UA versus controls and AMI versus controls, as all interfering factors were disease-associated factors, no factor needed to be corrected. As a second random run (the first random run was the classification before correction) without correcting any factors was selected as a negative control to compare with correction of interfering factors, unlike that in interfering-factor correction runs, an *n* value of 22 and a *p* value of 0.7 in UA versus controls and an *n* value of 18 and a *p* value of 0.7 in AMI versus controls were selected as the best parameter combinations for downstream analysis because of the highest AUC in classifying disease and control samples in the second random no-correction run. NMDS and ANOSIM showed no difference between cases and controls (Fig. S4A to D).

### Identification of gut microbial indicators associated with different CAD groups after noise correction.

With corrected CLR values, microbiota indicators were identified using the R package indicspecies for case/control samples (see Materials and Methods). After indicator identification, MDA of each indicator in the 100-permutation training set for case/control classification after correction was extracted according to the selected *n*-*p* parameter combination. Besides MDA, the CLR value difference of each indicator was also acquired with a one-tailed *t* test for confidence validation.

**FIG 4:**
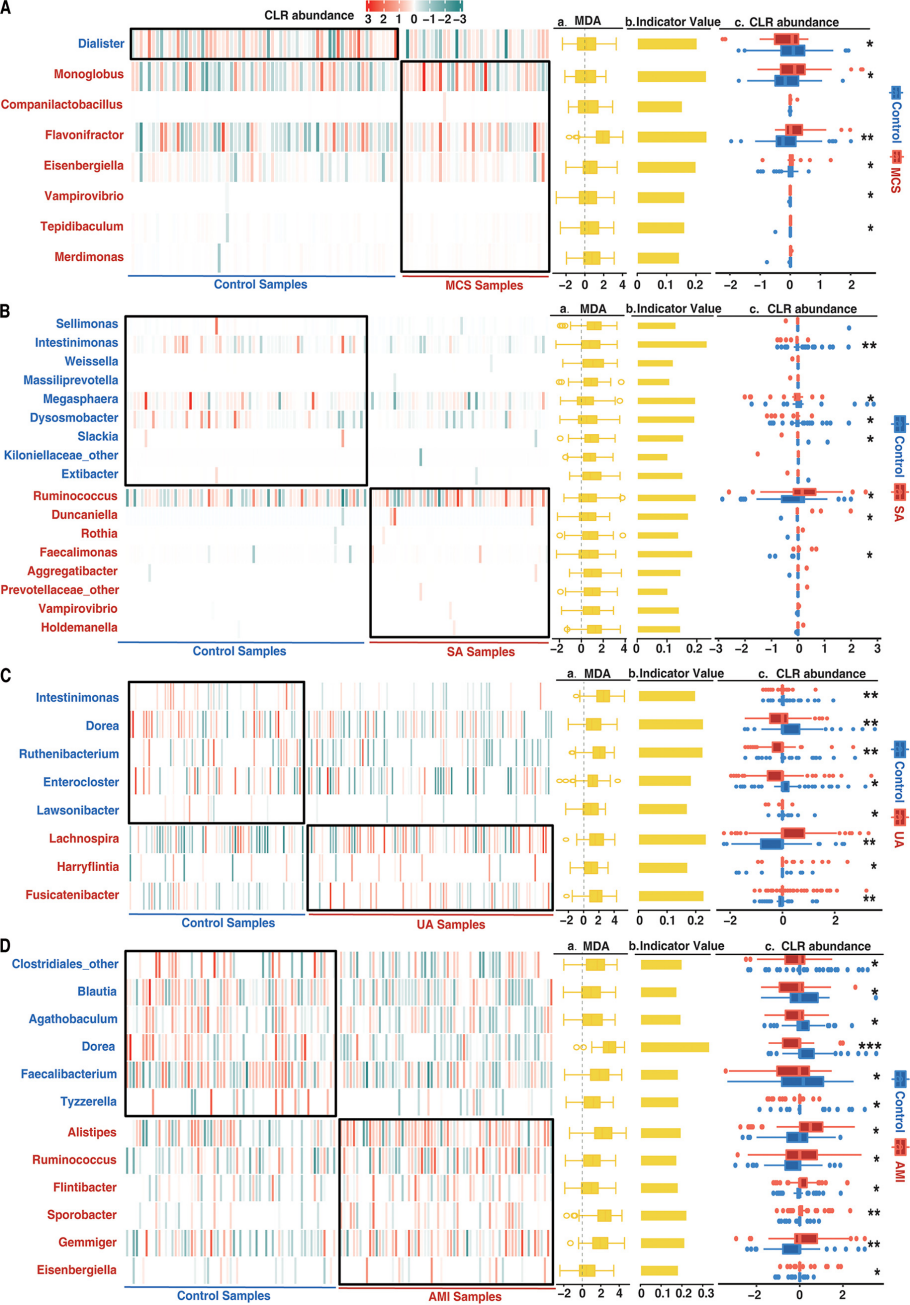
Overall abundance difference of case or control indicators in (A) MSC versus controls, (B) SA versus controls, (C) UA versus controls, and (D) AMI versus controls. The main panels show heat map-complex plots (heat maps combined with annotating plots [panel a to c]) for 4 groups. In each heat map-complex plot, the heat map was plotted for the indicator genus identified from a CLR-transformed and corrected abundance table which yielded the highest AUC elevation in classification of case/control samples. (a) MDA values of indicator genera are presented in the same rows as in the heat map on the left. MDA values were extracted from the training set in 100 permutations for importance indication. (b) Indicator values of the indicator genera in the same rows in the heat map on the left. (c) Differences in CLR-transformed and corrected abundance of the indicators in the same rows in the heat map on the left between cases and controls explored with the one-tailed *t* test. Indicators were identified with the R package indicspecies. *, 0.01 < *P* < 0.05; **, 0.001 < *P* < 0.01; ***, 0.0001 < *P* < 0.001; ****, *P* < 0.0001.

In MCS versus controls after correction of age, the genus *Dialister* (family *Veillonellaceae*, phylum *Firmicutes*) was identified as a control indicator, and seven bacterial genera were identified as the MCS indicators (*P* < 0.05) (Fig. 4A), including three genera (*Monoglobus*, *Flavonifractor*, and *Tepidibaculum*) from the family *Oscillospiraceae* in the phylum *Firmicutes*, three genera (*Companilactobacillus*, *Eisenbergiella*, and *Merdimonas*) from the family *Lactobacillaceae* in the phylum *Firmicutes*, and one genus (*Vampirovibrio*) from the order *Vampirovibrionales*, phylum *Firmicutes*. The significance of the indicators was confirmed by MDA (Fig. 4A, panel a), indicator values (Fig. 4A, panel b), and one-tailed *t* test (Fig. 4A, panel c; Table S9).

After the correction of BMI in SA versus controls, a total of eight bacterial genera were identified as SA indicators (Fig. 4B), including *Ruminococcus* (family *Oscillospiraceae*, phylum *Firmicutes*), *Duncaniella* (family *Muribaculaceae*, phylum *Bacteroidetes*), *Rothia* (family *Micrococcaceae*, class *Actinomycetia*), *Faecalimonas* (family *Lachnospiraceae*, phylum *Firmicutes*), *Aggregatibacter* (family *Pasteurellaceae*, phylum *Gammaproteobacteria*), *Vampirovibrio* (family *Vampirovibrionales*, phylum *Firmicutes*), *Holdemanella* (family *Erysipelotrichaceae*, phylum *Firmicutes*), and unclassified *Prevotellaceae* (phylum *Bacteroidetes*). Another nine genera were identified as control indicators (Fig. 4B), including three genera *Sellimonas*, *Weissella*, and *Extibacter* from the family *Lachnospiraceae* in the phylum *Firmicutes*, *Intestinimonas* (*Eubacteriales incertae sedis*, phylum *Firmicutes*), *Massiliprevotella* (family *Prevotellaceae*, phylum *Bacteroidetes*), *Megasphaera* (family *Veillonellaceae*, phylum *Firmicutes*), *Dysosmobacter* (family *Oscillospiraceae*, phylum *Firmicutes*), *Slackia* (family *Eggerthellaceae*, phylum *Actinobacteria*) and unclassified *Kiloniellaceae* (phylum *Alphaproteobacteria*). A median MDA much higher than 0 (Fig. 4B, panel a) and indicator values (Fig. 4B, panel b) as well as the significance of the single *t* test further confirmed the indicator significance (Fig. 4B, panel c). *Vampirovibrio* was an identical disease indicator which was identified in both MCS and SA comparing with control (Fig. 4A and B; Table S10).

In the comparison of UA versus controls without correction, five genera were identified as control indicators, including *Intestinimonas* (*Eubacteriales incertae sedis*, phylum *Firmicutes*), *Lawsonibacter* (*Eubacteriales incertae sedis*, phylum *Firmicutes*), *Dorea* (family *Lachnospiraceae*, phylum *Firmicutes*), *Enterocloster* (family *Lachnospiraceae*, phylum *Firmicutes*), and *Ruthenibacterium* (family *Oscillospiraceae*, phylum *Firmicutes*). Three genera, *Lachnospira* (family *Lachnospiraceae*, phylum *Firmicutes*), *Fusicatenibacter* (family *Lachnospiraceae*, phylum *Firmicutes*), and *Harryflintia* (family *Oscillospiraceae*, phylum *Firmicutes*), were identified as UA indicators. *Intestinimonas* was identified as a control indicator when controls were compared with both SA and UA (Fig. 4B and C; Table S11).

In the comparison of AMI versus controls, six genera were identified control indicators, including an unidentified *Clostridiales* genus (order *Clostridiales*, phylum *Firmicutes*), *Blautia* (family *Lachnospiraceae*, phylum *Firmicutes*), *Dorea* (family *Lachnospiraceae*, phylum *Firmicutes*), *Tyzzerella* (class *Clostridia*, phylum *Firmicutes*), *Agathobaculum* (family *Oscillospiraceae*, phylum *Firmicutes*), and *Faecalibacterium* (family *Oscillospiraceae*, phylum *Firmicutes*). Six genera were identified as AMI indicators, including *Alistipes* (family *Rikenellaceae*, phylum *Bacteroidetes*), *Ruminococcus* (family *Oscillospiraceae*, phylum *Firmicutes*), *Flintibacter* (*Eubacteriales incertae sedis*, phylum *Firmicutes*), *Sporobacter* (family *Oscillospiraceae*, phylum *Firmicutes*), *Gemmiger* (*Eubacteriales incertae sedis*, phylum *Firmicutes*), and *Eisenbergiella* (family *Lachnospiraceae*, phylum *Firmicutes*). Interestingly, *Dorea* was consistently identified as a control indicator of UA and AMI (Fig. 4C and D; Table S12). *Ruminococcus* belonged to the indicators of SA and AMI (Fig. 4B and D; Table S12). *Eisenbergiella* was an indicator of MCS and AMI (Fig. 4A and D; Table S12). Our discovery of indicators shared by different groups implies conserved roles of gut microbiota members possibly associated with CAD development.

To estimate the effectiveness of noise correction for identification of indicators, we compared the case/control differences of indicator microbiota organisms before and after noise correction. Significant differences for most indicator bacteria were observed in comparisons of MCS versus controls before and after correction (Fig. S5A). Few changes were found in other groups (Fig. S5A). This suggests that noise correction was important for identifying MCS-associated microbial indicators.

### Differential diagnosis of clinical indexes among four CAD groups.

Here, we estimated diagnostic difference of clinical indexes in each CAD group with the Wilcoxon test for significance and the Benjamini-Hochberg method for multiple-comparison adjustment. The detailed clinical indexes for the diagnosis of CAD included fasting blood glucose (FBG), glycosylated hemoglobin (HbAlc), alanine transaminase (ALT), aspartate amino transferase (AST), blood urea nitrogen (BUN), cholesterol (CHOL), creatine kinase (CK), creatine kinase-MB (CK-MB), creatinine (CR), cardiac troponin-I (cTn.I), hemoglobin (Hb), hydroxybutyrate dehydrogenase (HBDH), lactate dehydrogenase (LDH), LDH1, and triglycerides (TG).

In the comparison of MCS versus controls, there was no significant difference among clinical indexes except age (Benjamini-Hochberg-adjusted *P* < 0.05, Wilcoxon test). In SA versus controls, age (*P* < 0.05, Wilcoxon test), CK (*P* < 0.05, Wilcoxon test), and CK-MB (Benjamini-Hochberg-adjusted *P* < 0.05, Wilcoxon test) were significantly higher in SA samples, while there was no significance in difference for other clinical indexes. For UA versus controls, besides age, CK and CK-MB, FBG, HBDH and TG also increased significantly in UA compared with controls (*P* < 0.05, Wilcoxon test). The difference in CK (*P* < 0.05, Wilcoxon test) and CK-MB (Benjamini-Hochberg-adjusted *P*< 0.05, Wilcoxon test) between cases and controls were even more significant than that in SA cases versus controls. In AMI versus controls, significant differences were found in 9 of 13 clinical indexes. Besides FBG, CK, CK-MB, HBDH and TG, significantly higher ALT (*P* < 0.05, Wilcoxon test), AST (Benjamini-Hochberg-adjusted *P* < 0.05, Wilcoxon test), BUN (Benjamini-Hochberg-adjusted *P* < 0.05, Wilcoxon test), LDH (Benjamini-Hochberg-adjusted *P* < 0.05, Wilcoxon test), and LDH1 (Benjamini-Hochberg-adjusted *P* < 0.05, Wilcoxon test) were found in AMI samples than in control samples. The differences in FBG (*P* < 0.05, Wilcoxon test), CK (Benjamini-Hochberg-adjusted *P* < 0.05, Wilcoxon test), CK-MB (Benjamini-Hochberg-adjusted *P* < 0.05, Wilcoxon test), HBDH (Benjamini-Hochberg-adjusted *P* < 0.05, Wilcoxon test) were much more significant than in UA cases versus controls, while TG did not show statistically significant elevation (Fig. S5B). In short, only one and three of 15 clinical indexes showed significant case-control differences in MCS and SA, respectively. Many more clinical indexes were significantly different in UA (6/15) and AMI (10/15) in comparison with controls. Our results suggest that most clinical indexes are reliable or useable only in later diagnosis of CAD. Thus, the development of novel potential markers is indispensable for the early diagnosis of CAD.

### Estimation of diagnostic potential with microbiota indicators in CAD patients.

**FIG 5:**
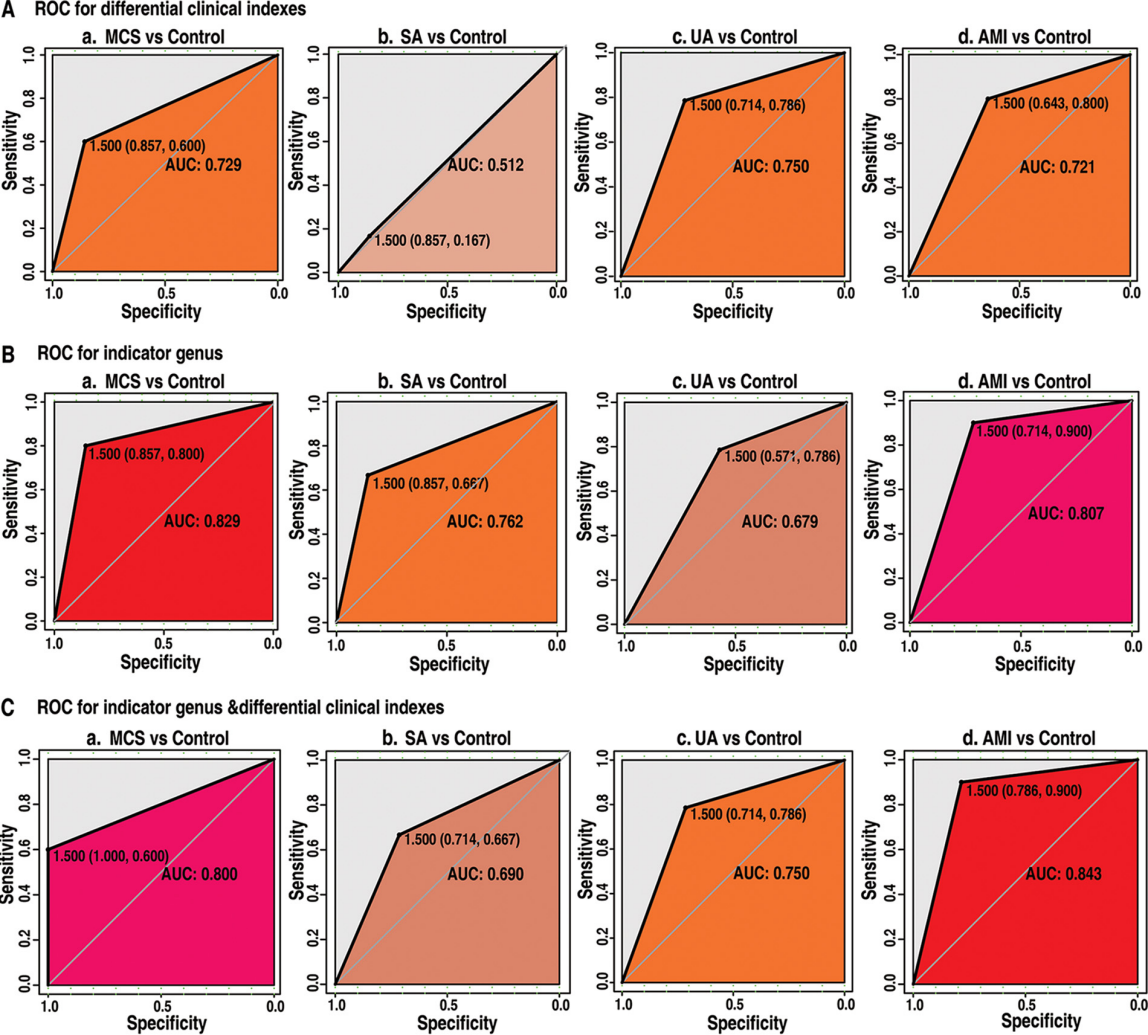
Receiver operating characteristic (ROC) curves of diagnostic models with differential clinical indexes and indicator genera using the random forest model. (A to C) ROC curves for significantly differential clinical indexes (A), indicator genus (B), and indicator genus and differential clinical indexes (C) versus controls; MCS (a), SA (b), UA (c), and AMI (d). Background color was correlated with AUC value; darker red indicates a higher AUC. Clinical indexes were used for this analysis that showed significant difference between case and control samples with Benjamini-Hochberg adjusted *P* value <0.05.

Clinical indexes were usually necessary for disease diagnosis. To estimate the diagnostic potential of microbiota indicators, using indicator bacteria or/and differential clinical indexes after carrying out Benjamini-Hochberg adjustments, we established disease diagnostic models for each group. Diagnostic models based solely on clinical indexes returned distinct AUC values of 0.729, 0.512, 0.750, and 0.721 in MCS versus controls, SA versus controls, UA versus controls, and AMI versus controls, respectively (Fig. 5A). In models relying solely on indicator bacteria, the AUC values for model accuracy were 0.829, 0.762, 0.679, and 0.827, respectively, for MCS versus controls, SA versus controls, UA versus controls and AMI versus controls (Fig. 5B). The modeling combined with both microbial indicators and clinical indexes returned AUC values 0.800, 0.690, 0.750, and 0.843 in MCS versus controls, SA versus controls, UA versus controls, and AMI versus controls (Fig. 5C). In summary, the highest AUC values were obtained by relying solely on indicator bacteria for diagnosis of both MCS and SA. Indicator bacteria cannot increase the AUC value of diagnostic accuracy in UA but can improve the diagnostic accuracy of AMI with clinical indexes. Gut microbiota as an indicator showed diagnostic potential.

### Correlation between the clinical indexes and gut microbial indicators.

Using linear regression analysis, we performed correlation analysis between gut microbial indicators (Fig. 4) and differential clinical indexes (Fig. S5B). Our results revealed 8, 2, 3, and 25 significant correlated pairs in MCS versus controls (Fig. S6A), SA versus controls (Fig. S6B), UA versus controls (Fig. S6C), and AMI versus controls (Fig. S6D), respectively.

**FIG 6:**
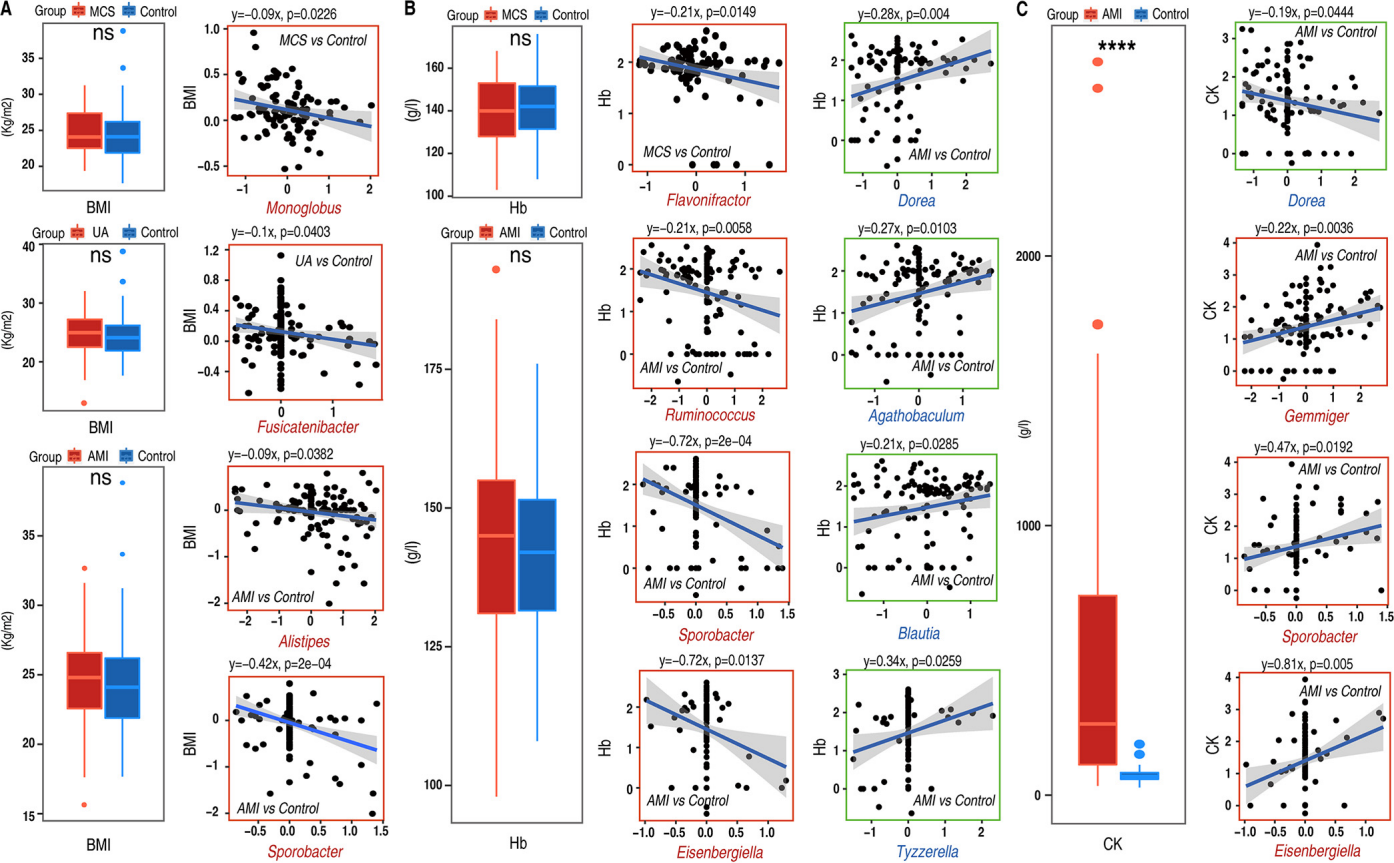
Identical correlation patterns between indicator genus and clinical indexes among different groups. (A) Identical correlation pattern between indicator genus and BMI among groups. The box plots on the left compare BMI values between case and control samples in groups where identical indicator-BMI correlation patterns were found. The boxes in right portion show the correlations of indicator genus with BMI in groups where the red boxes and red x axis labels indicate that the BMI were significantly associated with disease indicator genera. (B) Identical correlation pattern between indicator genus and Hb. The box plots on the left show the difference in Hb values between case and control samples in groups where identical correlation patterns with Hb were found. Graphs in the middle show the identical pattern in interaction between disease indicator genus and Hb. The red boxes and red *x* axis labels indicate that the interactions with Hb were disease indicator genus associated. Graphs on the right show the identical pattern in interaction between control indicator genus and Hb, in which green boxes and blue *x* axis labels indicate that the interactions with Hb were control indicator genus associated. (C) Identical correlation pattern between indicator genus and CK. The graph on the left shows the comparison of CK values in AMI versus controls where the identical interaction pattern was found. Graphs on the right show the identical but opposite pattern in interaction of CK with control and disease indicator genus, in which green boxes and blue *x* axis labels indicate the control indicator-associated interaction, while red boxes and red *x* axis labels indicate the disease indicator-associated interaction with CK. ****, *P* < 0.0001; ns: no significance (Wilcoxon test).

Our results indicated that 12 microbial indicators from four disease groups showed significant and consistent patterns of association with the same clinical indexes. For example, although no significant BMI differences were found in MCS, UA, and AMI compared with control samples (Fig. 6A), BMI showed significantly consistent negative correlations with disease indicators such as the MCS indicator *Monoglobus* (Fig. 6A), the UA indicator *Fusicatenibacter* (Fig. 6A), and the AMI indicators *Alistipes* and *Sporobacter* (Fig. 6A). When we normalized the FBG values, there were no significant associations between FBG and indicator genus in 4 groups. However, FBG without normalization showed significantly negative associations with the MCS indicator *Merdimonas* and the control indicator (versus SA) *Megasphaera* (Fig. S6E). Though Hb showed no significant case/control difference in MCS versus controls and AMI versus controls (Fig. 6B), the MCS indicator *Flavonifractor* (Fig. 6B) and the AMI indicators *Ruminococcus*, *Sporobacter*, and *Eisenbergiella* (Fig. 6B) all showed consistent negative interplay with Hb. At the same time, the control indicators (in AMI versus controls) *Blautia*, *Agathobaculum*, *Dorea*, and *Tyzzerella* all showed significantly consistent positive associations with Hb (Fig. 6B). Another clinical index, CK, which was significantly increased in AMI compared to control samples (Fig. 6C), was found to be negatively associated with the control indicator (in AMI versus controls) *Dorea* and positively associated with the AMI indicator *Sporobacter*, *Gemmiger* and *Eisenbergiella* consistently (Fig. 6C). It was interesting that in AMI versus controls, the control indicator *Dorea* and the AMI indicators *Sporobacter* and *Eisenbergiella* all showed significant associations with both CK and Hb identically and with opposite trends (Fig. 6B and C). Meanwhile, *Sporobacter* also showed a negative association with BMI in AMI patients. Such consistent and opposite trends provide further information about the potential role of gut microbiota in development, diagnosis, or therapy of CAD.

## DISCUSSION

Multiple CAD risk factors affect gut microbiota compositions in CAD patients, and the diverse composition of the gut microbiota results in different responses to pathophysiology in the cardiovascular system (31); however, whether the effect of a specific risk factor on the gut microbiota contributed to pathophysiology was uncertain. In this study, using a random forest classifier and PCA-based method, we identified the disease- and noise-associated roles of several interfering factors from the confounding factors, estimated the degree of influence, removed confounding noises, and gained deeper insight into the relationship between CAD clinical indexes and gut microbes.

With our methods, the CAD risk factors gender, hypertension, age, and BMI showed a high degree of interference in the gut microbiotas of CAD patients. For the 4 interfering factors identified, according to AUC changes, BMI in MCS versus controls and UA versus controls, age in UA versus controls and AMI versus controls, hypertension in SA versus controls and AMI versus controls, and gender in UA versus controls likely play important roles in disease development by interfering with gut microbiota, while the interference in gut microbiota by BMI likely contributed little to disease development in SA versus controls.

It was interesting to find that BMI plays a disease-associated role in MCS and UA but not in SA, as the degree of interference in the gut microbiota explained by BMI in SA versus controls was the highest (Fig. 2D) and higher than that in MCS versus controls or in UA versus controls. It is well known that obesity or abundant blood lipid can promote the formation of atherosclerosis (32), and a disturbed gut microbiota due to high BMI level further induces multiple proinflammatory activities or produced metabolites to accelerate MCS development (33). However, for SA patients, the “obesity paradox” was discussed, as obese patients usually showed better clinical outcome than lower-BMI patients (34). In patients with vasospastic angina, obesity usually tends to be associated with favorable 1-year primary endpoints (35), while lower-BMI patients with suspected stable angina pectoris showed a higher risk of AMI and cardiovascular (CV) death (36). Few studies have reported on this mechanism, in which BMI is assumed to be involved in unknown or unconsidered pathways in SA patients (37). It was found that the contribution of BMI interference in gut microbiota to disease development was different at least in MSC and SA patients. It was also noted that the same confounding factor (for example, BMI) may play different roles in different disease groups in CAD patients.

As age was a risk factor for CAD and a known gut microbiota-interfering factor, it is not surprising that its interference in gut microbiota was associated with development of UA and AMI. However, age was not confirmed to have a disease-associated role in MCS and SA patients according to our results. As men age, functional and electrical defects appear in the heart, and other factors, such as a high level of inflammation and elevated oxidative stress, especially for the production of reactive oxygen species (ROS) associated with persistent inflammation, also occur with high prevalence (38). The gut microbiota changes as the host ages, combined with a decrease in beneficial organisms like *Bifidobacterium*, which could scavenge ROS, and an increase in proinflammatory commensal microbes. At the same time, the proinflammatory alteration could be reversed by fecal microbiota transplant (FMT) from young donors (39). As inflammation plays pivotal roles in CAD development (40), CAD interference in the gut microbiota in advancing age likely makes a significant contribution.

Hypertension interference in the gut microbiota was found, showing its disease-associated roles in SA and AMI patients. Hypertension was one known major risk for CAD and also showed close connections with gut microbes. On the one hand, short-chain fatty acids (SCFA) with anti-inflammation functions produced by gut microbes bind receptors expressed in the kidney to stimulate renin secretion and activate the renin-angiotensin-aldosterone system (41), which plays crucial roles in sodium handling and salt sensitivity control, to increase blood pressure. On the other hand, gut microbes (for example, lactobacilli) could inhibit ACE1 by production of active peptides, leading to lower production of angiotensin II, playing a protective role in blood pressure control (42).

Gender, which was a risk factor for CAD, was also found to play disease-associated roles in the gut microbiota in UA versus controls. Sex-specific differences in CAD development and gut microbiota composition are widely reported in animal (43) and human (44) studies. Sex hormones influence the gut microbiota, and due to the effects on cholesterol rates and redirection of lipoproteins toward low-density lipoprotein (LDL) of sex hormones, men show a higher CAD risk, while women are more prone to suffer CAD at older ages, when estrogen production ceases in menopause, and they show much higher mortality (45).

Among risk factors identified here, some were responsible only for noise, while contributions to CAD development of some were bridged by interference in the gut microbiota, and even for the same factor, different roles in CAD development were found here. Specifying the roles of each factors interfering the gut microbiota was useful not only for noise controlling but also for better interpreting the linkages between gut microbes and disease development, which was also reflected in our results.

Though MCS, SA, UA, and AMI appear to be different CADs, the identification of identical disease or control indicators by comparing with the same controls may also suggest some gut microbiota similarities in CAD patients. Many indicator genera have been reported in published articles after indicator analysis. In MCS versus controls after correction of age noise, the control indicator *Dialister*, a genus in the phylum *Firmicutes*, was significantly depleted in Tibetan coronary heart disease (CHD) patients compared to healthy Tibetans and showed negative associations with lipopolysaccharide (LPS), consistent with its protective anti-inflammation role (46). Most MCS indicators were found to be from the families *Oscillospiraceae* and *Lactobacillaceae*, usually playing favorable roles in CAD patients for production of butyrate (47). It worth noting that the MCS indicators *Monoglobus* (47) and *Flavonifractor* (48) were significantly decreased in CAD patients in other studies. Some studies reporting that degradation of flavonoids by Flavonifractor plautii contributed to human health by exerting an anti-inflammatory effect (49). The detection of likely beneficial roles for MSC indicators after correction of age noise led to the hypothesis that some gut microbes probably play certain protective roles in adaptation to the transition of MCS status.

In SA versus controls, after correction of BMI noise, the control indicator *Intestinimonas*, which was identified in control samples when they were compared with both SA and UA, was reported to be negatively associated with saturated fatty acids, which serve as a biomarker in overweight/obese subjects (50). Among SA indicators, *Ruminococcus* was positively correlated with high-protein and -fat diets (51) and higher TMAO production (52) and was reported to be positively associated with type 2 diabetes mellitus (T2DM), chronic heart failure and atrial fibrillation (AF), playing a proinflammatory role with cytokine production (53). Bacteria from the genus *Rothia* were significantly enriched in patients with isolated diastolic hypertension (IDH) and isolated systolic hypertension (ISH) (54), which are high-risk factors for CAD. Species from the genus *Aggregatibacter* were found to promote the formation of atherosclerosis plaques after oral or intravenous infection (55). With correction of BMI, some controversy also exists. For example, *Megasphaera* was reportedly characterized with higher level in AMI samples (56) but was identified as a control indicator in our study; this might be related to either the SA-AMI difference or the BMI noise correction, which also indicated the necessity to explore the meaningful roles of gut microbes after noise identification and correction.

In UA versus controls without correction, control indicator organisms of the genus *Dorea* were reported at lower levels in heart failure patients (57) and negatively associated with N-terminal pro-B-type natriuretic peptide (NT-proBNP) values in CAD patients (58). As NT-proBNP was a powerful biomarker for heart failure, as its levels were reported to be positively associated with CAD severity (59), the potential protective role of *Dorea* in CAD development was proved. The genus *Fusicatenibacter* was identified as a UA indicator in comparison with control samples. *Fusicatenibacter* is a butyrate-producing genus and plays an important role in maintaining gut health. Aside from its anti-inflammation role (60), *Fusicatenibacter* can also modulate colonic motility via butyrate (61) and alleviate constipation symptoms by short-chain fatty acids as well as hormone secretion (62). However, as discussed above, increased SCFA can increase blood pressure by stimulating renin secretion (41), and *Fusicatenibacter* showed a significant association with systolic blood pressure and diastolic blood pressure in hypertension patients (63). More studies of the associations between butyrate-producing bacteria and CAD are needed.

In AMI versus controls without correction, all control indicators were from the families *Lachnospiraceae* and *Oscillospiraceae*. Besides *Dorea*, which was identified as a control indicator in UA versus controls and in AMI versus controls, another *Lachnospiraceae* genus, *Blautia*, has been reported to have a protective role, being associated with decreases in atherosclerotic lesions, lower concentrations of tumor necrosis factor alpha (TNF-α) and interleukin 1β (IL-1β), and lower plasma total cholesterol (64–66). The *Oscillospiraceae* family genus *Faecalibacterium* is also a butyrate-producing genus and was identified as a control indicator; its hypertension-associated role may be slight. Two AMI indicators, *Alistipes* and *Sporobacter*, were believed to contribute to inflammation as well as epithelium alterations in hypertension patients (67, 68). The genus *Alistipes* was also found to have a significant and positive association with serum dimethylglycine, whose level is closely correlated with mortality in CAD patients (69); *Gemmiger* was also reported as a significant genus in CAD patients (70), and the SA indicator *Ruminococcus* was also identified as an AMI indicator, all of which suggest disease roles in AMI.

To estimate the diagnostic potential of gut microbes, we established and compared diagnostic models by applying indicator bacteria and/or differential clinical indexes. Distinct prediction performances were found in four groups. For MCS versus controls, the highest AUC value was 0.829, solely returned by microbial indicators. Similarly, the highest AUC value of 0.762 was also returned by microbial indicators alone for SA versus controls. These results indicated the better diagnostic potential for gut microbes in early-stage CADs. For UA versus controls, the consistent highest AUC value, 0.75, was obtained according to the clinical indexes or the combination of clinical indexes. This finding indicated that microbial indicator cannot improve the UA diagnosis based on clinical indexes. For AMI versus controls, the AUC values were 0.843 (clinical indexes and microbial indicators), 0.807 (microbial indicators), and 0.721 (clinical indexes). This result suggested that both microbial indicators and clinical indexes could return the best diagnostic performance, suggesting that the gut microbiota can increase the diagnosis resolution of AMI based on clinical indexes (Fig. 5C) and showing its potential for CAD diagnosis.

In a correlation analysis between indicator genus and clinical indexes, though there was no significant difference in BMI in MCS versus control patients, the indicator *Monoglobus* showed significantly negative linkage with BMI. The same negative association was reported in another study in individuals with high atherosclerotic cardiovascular disease risk; however, when low-calorie dietary interventions were performed, atherosclerotic markers improved in a time series study (47). When mice were orally administered Qing-Xin-Jie-Yu Granule (QXJYG), a Chinese traditional decoction which was proven to be clinically effective for CAD patients, *Monoglobus* was also significantly elevated (71). Considering that the results in our study for MCS versus controls were obtained after correction of age noise (and it is critical to note that age influence was also experimentally removed in both studies for samples collected from the same individual or model mice), they indicated that the correlation between *Monoglobus* and BMI might be inherent and further make the protective role of *Monoglobus* clearer, as higher BMI was reported to be associated with higher inflammation and higher CAD development risk, especially for MCS patients (72).

Though *Monoglobus* was just one indicator, as a high-abundance indicator, its negative linkage also led to an assumption that some MCS gut microbes might play protective roles in MCS patients, which resulted from the response to the selection of host adaptation in the early stage of CAD development during the transition from a non-CAD to a CAD state. However, the protective power was made less clear by gut microbiotas with or without host influence induced by age in our study of the significance of age difference between MCS patients and controls.

Aside from the negative association between MCS indicators and BMI, UA and AMI indicators also showed a significant negative connection with BMI. For healthy individuals, higher BMI was associated with higher CAD risk; however, as discussed previously, advanced-CAD patients with higher BMI usually showed better clinical outcome (73). It is logical to speculate that the highly abundant UA indicator *Fusicatenibacter* and the AMI indicators *Alistipes* and *Sporobacter* were assumed to indicate a worse prognosis for UA and AMI patients according to the negative association with BMI. As discussed above, the UA indicator *Fusicatenibacter* as well as the AMI indicators *Alistipes* and *Sporobacter* showed associations with hypertension. In addition, *Alistipes* shared positive connection with mortality in CHD patients (69).

Similarly, FBG shared negative linkages with the MCS indicator *Merdimonas* and another control indicator (versus SA), *Megasphaera*. For FBG, which is associated with the risk and severity of heart disease (74, 75), it is not surprising to find the negative connection of FBG with the control indicator (versus SA) *Megasphaera*. However, it is interesting to find a negative correlation between FBG and the MCS indicator *Merdimonas*. As discussed with regard to the BMI correlation analysis, MCS indicators may likely play a favorable role in response to transition of MCS status from a healthy state. The MCS indicator *Merdimonas*, which is negatively correlated with FBG, may represent the beneficial role of gut microbes in MCS patients.

Besides identical patterns of correlation of BMI and FBG with disease indicators, identical patterns of correlation of Hb with indicators for different CADs also attracted our attention. Anemia or low hemoglobin concentration leads to higher fibrinogen levels (76) in plasma and contributes to myocardial ischemia development (77), acting as a risk factor for adverse outcome in CAD patients (78). Considering that Hb values in MCS and AMI samples were in the normal or lower range (13 to 18 g/dL for men; 12 to 16 g/dL for women) and showed no significant differences in comparisons of MCS versus controls and AMI versus controls, identical negative associations with Hb for the MCS indicator *Flavonifractor* and the AMI indicators *Ruminococcus*, *Sporobacter*, and *Eisenbergiella* as well as identical positive associations with Hb for control indicators in AMI versus controls (*Blautia*, *Agathobaculum*, *Dorea*, and *Tyzzerella*) suggested the diagnostic potential of gut microbes together with Hb in CAD patients.

In addition to BMI and Hb, negative connections of CK values with the control indicator *Dorea* and identical positive associations with *Sporobacter*, *Gemmiger*, and *Eisenbergiella* were also found, with similar trends. It was noted that *Dorea*, *Sporobacter*, and *Eisenbergiella* showed opposite but identical correlations with both Hb and CK in AMI versus controls, suggesting the potential therapeutic and diagnostic potential of the 3 gut microbes in AMI patients. Altogether, BMI-, HB-, and CK-associated genera might constitute candidate targets for potential diagnosis or interventions of CAD development in a more specific way.

In conclusion, we present more specific insight into the alteration in the gut microbiota between different CAD and control samples, with identification of disease-associated interfering factors and elimination of background noise, and we further reveal diverse associations between gut microbes and clinical markers, which might contribute to the CAD development or severity in the targeted populations. With the establishment of diagnostic models, the identical patterns of some gut microbes’ correlation with development of different diseases may lead to more attention being focused on studying them for disease diagnosis or treatment of CAD patients.

## MATERIALS AND METHODS

### Patient recruitment.

To explore the shift of the gut microbiota in different stages of CAD, 306 patients who were diagnosed as having mild coronary stenosis, stable angina, unstable angina, or acute myocardial infarction as well as non-CAD patients at the Department of Cardiology, Yan’an Affiliated Hospital of Kunming Medical University, in Kunming were recruited in our study according to 2020 ESC guidelines for the management of CAD (30), as follows: (i) MCS is defined as luminal narrowing in the rage of <50% by coronary angiography; (ii) SA is a disease causing exercise- and stress-related chest symptoms due to narrowing of more than 50% in the left main coronary artery and more than 70% in one or several of the major coronary arteries; (iii) UA is defined as myocardial ischemia at rest or on minimal exertion in the absence of acute cardiomyocyte injury/necrosis with the non-ST (S-wave and T-wave) segment in electrocardiogram; (iv) AMI is defined as acute chest pain and cardiomyocyte necrosis, including ST segment elevation myocardial infarction (STEMI) and non-ST-segment elevation myocardial infarction (NSTEMI); (v) non-CAD patients were recruited as controls if the participant was diagnosed as being free of coronary artery disease.

### Ethics approval and consent to participate.

The study was approved by the ethics committee of Yan’an Affiliated Hospital of Kunming Medical University. Informed consent was obtained from involved patients or their guardians.

### Sample collection.

Fresh fecal samples were collected from the first defecation in the morning. Samples were collected by the participants or collected from diapers by their guardians. All samples were immediately frozen and stored below −20°C before being sent to the laboratory, where samples were stored at −80°C in tubes with DNA protection solution.

### 16S RNA sequencing.

Gut microbiota composition in each sample were determined by 16S sequencing. Bacterial DNA was extracted using an E.Z.N.A. stool DNA kit (Omega Bio-tek, Norcross, GA, USA) according to the protocol. Then, the V3-V4 regions of the bacterial 16S rRNA gene were PCR amplified with primers (338F, 5′-ACTCCTACGGGAGGCAGCAG-3′; 806R, 5′-GGACTACHVGGGTWTCTAAT-3′). Finally, the pooled purified amplicons were sequenced in 250-bp paired-end runs on the Illumina MiSeq platform (Illumina, San Diego, CA, USA).

### 16S sequencing data preprocessing and analysis.

Raw 16S sequences were demultiplexed using qiime2 (79) and denoised using DADA2 (80), including filtering low-quality score bases, removing chimeric sequences and singletons, joining denoised paired-end reads, and dereplicating sequences. The amplicon sequence variant (ASV) table was finally obtained, and the rarefaction curves of species richness were plotted. According to the lowest saturation abundance for richness among all samples, the ASV table was subsampled at an even depth of 10,000. Taxonomy classification was performed with pretrained naive Bayes classifiers on Silva_132_99%_OTUs_full-length sequences.

### PCA-associated method used to denoise confounding factors.

Briscoe and colleagues found that the centered log ratio (CLR) transformation of microbiome data combined with an unsupervised principal-component correction approach showed an ability to reduce confounding noises comparable to that of supervised approaches (29). To eliminate the interference of confounding factors on the actual difference between the controls and patients in gut microbiome composition as much as possible, we utilized the principal-component analysis (PCA) method from the work of Briscoe et al. (29) and made some modifications for background noise detection and correction. In our study, empirical confounding diseases or factors which accompanied samples in different groups were different and inconsistent. Consequently, whether a factor was involved in disease development or represented noise was also inconsistent in different groups. Thus, to identify the microbes involved in disease development in different groups, it was necessary for us to identify confounding factors and carry out associated noise correction as well as the following comparison together with control samples separately for each disease group. It was noted that Briscoe and colleagues (29) regressed the top *p* PCs and the optimized *p* was validated according to AUC estimated by random forests. However, in our data, the top PCs usually accounted for the major variance for case-control comparison, and therefore, regressing out the top PCs may be somewhat arbitrary. Thus, it was more appropriate for us to regress just the target binary confounding factor-associated PCs, which was validated by the random forest approach according to AUC, or PCs significantly associated with continuous confounding factors given by GLM (see the flowchart in Fig. S2).

In detail, at first, for the table of ASVs subsampled at a depth of 10,000, genus abundance lower than 10 was set as 0, and genera present in at least 2 samples were maintained, following which CLR transformation of the genus abundance table was performed. Calculation of PCs scores was performed using the pca_method function of Briscoe et al. (29), which is publicly downloadable (https://github.com/garudlab/Microbiome_PCA_correction). To calculate PCA scores more conservatively, we set the numbers of PCs to calculate as *n*, and *n* ranged from 5 to 30. After *n* PCs scores were calculated, a metadata file containing all confounding factors, such as age, BMI, and gender, and a total of 24 confounding diseases, including type 2 diabetes, fatty liver, liver damage, and chronic gastritis, were matched with *n* PC scores according to sample ID. Then, the PC score table and metadata table of samples from the MCS, SA, UA, and AMI groups were extracted as case samples (*m*1 samples) and combined with control samples (*m*2 samples) separately as an input *m*×*n* PC score matrix with *m* rows of samples (*m*1 case samples + *m*2 control samples) and *n* columns of PCs (*n* PCs to calculate) and an *m*×*f* confounding factor matrix with *m* rows of samples and *f* columns of confounding factors for disease group depending on the operation process, including identification of confounding factor-associated PCs, the following confounding PC regression, AUC comparison between corrected and noncorrected values in case/control classification, selection of parameters with the best performance, and the composition comparison between cases and controls.

In each operation process, to identify the associated PCs for each discrete confounding factor (1 indicates presence and 0 indicates absence in the sample), the random forest method was applied in a “leave-one-data set-out” strategy with fraction *p* of samples selected as the training set (*p* for cases and *p* for controls) and the remaining sample set as the validation set. The smallest number of samples with confounding disease in all groups was 10, and because of the indeterminacy in the influence of sample count on better random forest performance, the fraction *p* was set as 0.5, 0.6, and 0.7. To avoid the randomness in each confounding-factor–PC association identification, sampling with fraction *p* and the leave-one-data set-out random forest method were performed 100 times. For each confounding factor, only confounding factors with AUC values of >0.5 in the validation set at least 80 times in 100 permutations were confirmed factors interfering with gut microbiota composition, and the associated PCs were confirmed with a mean decrease accuracy (MDA) value of >0 in the training set 80 times in 100 permutation. and for continuous factors, such as age and BMI, for which confidence is stronger with more samples, associations between PCs and confounding factors were identified using direct GLM (Poisson distribution) with the following formula: GLM(age/BMI ~ PC1+PC2…+PC*n*, family = Poisson(link = ‘identity’), where *n* is the number of PCs to calculate. The significance of factor-PC association was confirmed with *P* values of <0.05 after the Akaike information criterion (AIC) step. It was noted that factors interfering with gut microbiota composition were factors likely affecting gut microbiota composition, at least in the present case and control samples. Influence of interfering factors was quantified as sum(score of associated PCs)/sum(score of all PCs). However, whether a certain interfering factor constituted noise or might be involved in disease development could be further estimated according to AUC changes in classification of case and control samples.

After identification of PCs associated with confirmed interfering factors, including both discrete and continuous ones, a score matrix of *j* interfering-factor-associated PCs (*m*×*j*) were extracted from an *m*×*n* PC score matrix and regressed from the original *m*×*g* CLR-transformed genus abundance table as follows, according to the work of Briscoe et al. (29): X^m^*^g^ ~ β^j^*^g^P^m^*^j^ + ε^m^*^g^, where *X* is the original *m*×*g* CLR-transformed genus abundance matrix with *m* sample rows and *g* genus columns, matrix β is the coefficient matrix, matrix *Ρ* is the *m*×*j* interfering PC score matrix, and matrix ε is the *m*×*g* residue matrix (CLR values, *m* sample rows and *g* genus columns) resulting from the regression of *j* interfering-factor-associated PCs from the original CLR-transformed genus abundance matrix. The *m*×*g* residue matrix represents the corrected values for downstream analysis.

After correction of interfering factors, the random forest leave-one-data set-out strategy was performed for classification of case and control samples, with both corrected CLR values and original CLR values for the comparison of AUC before correction and after correction to estimate the performance of interfering factor correction. The same sample fraction was applied in classification of cases and controls in both CLR matrixes as in confounding-factor–PC association identification.

To determine the effect of each confirmed interfering factor alone on microbiota composition in each disease-dependent operation process mentioned above, metadata files which contained only the interfering factors were used for identifying associated PCs, correcting PCs, and estimating correction performance according to AUC changes in case control classification with original CLR values and corrected CLR values. With the same value of PCs (*n*) and the same sample fraction (*p*), a 100-permutation random forest method was performed with original and corrected CLR values for AUC comparison.

The noise-associated factors and disease-associated factors were also determined according to the AUC changes. For 100 permutations for each parameter combination (*n* and *p*), there were 100 AUC for original and corrected CLR values with which the Wilcoxon test was used for difference significance. The change in AUC was calculated using the median AUC. According to the AUC changes compared with AUC before correction, factors with significant AUC elevation after correction were considered noise-associated factors, while factors with significant AUC decrease after correction were considered disease-associated factors.

Considering that only noise needs to be removed, CLR values corrected with all noise factors were extracted, and the CLR values corrected with the parameter combination (*n* and *p*) with the highest fold change compared with uncorrected origin CLR values were used for indicator analysis between case and control samples; for samples in certain groups whose confounding factors were all disease-associated factors showing significantly decreased AUC in classifying case and control samples after correction, noncorrected original CLR values with a parameter combination giving the highest AUC (not fold change) were used for indicator analysis.

Alpha diversity, including richness and Pielou’s evenness, and beta diversity in each cluster were determined using the R package vegan. Indicator analysis of each CAD group in each cluster was performed using the R package indicspecies (81) at the genus level. Correlation between indicator genera with corrected CLR values and clinical indexes was performed by linear fitting with the Levenberg-Marquardt method (82).

### Data availability.

The raw data of 16S rRNA gene sequences are available at the NCBI Sequence Read Archive (SRA) with BioProject ID PRJNA779598.

## Supplementary Material

Reviewer comments
