# Supplementary material for: Diversified Shifts in the Cross Talk between Members of the Gut Microbiota and Development of Coronary Artery Diseases
Source: Microbiol Spectr. 2022 Oct 27;10(6):e02804-22. doi: 10.1128/spectrum.02804-22 (PMC9769841; doi:10.1128/spectrum.02804-22)
Supplement: Supplemental file 1 — Fig. S1-Fig. S2 and figure legends. Download spectrum.02804-22-s0001.pdf, PDF file, 6.6 MB [file spectrum.02804-22-s0001.pdf]

Figure S1

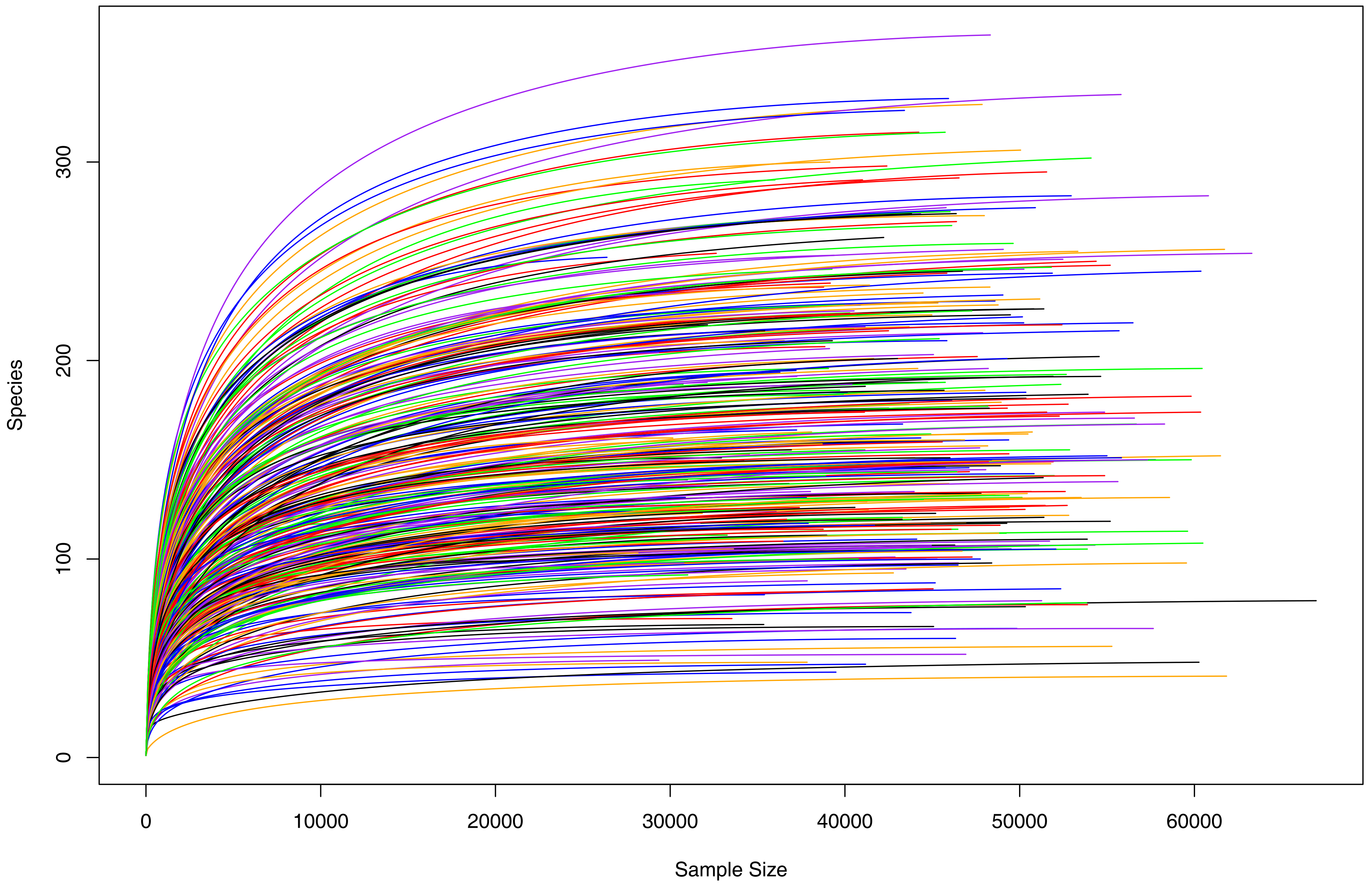
Species
Sample Size
0
100
200
300
0
10000
20000
30000
40000
50000
60000

Figure S2

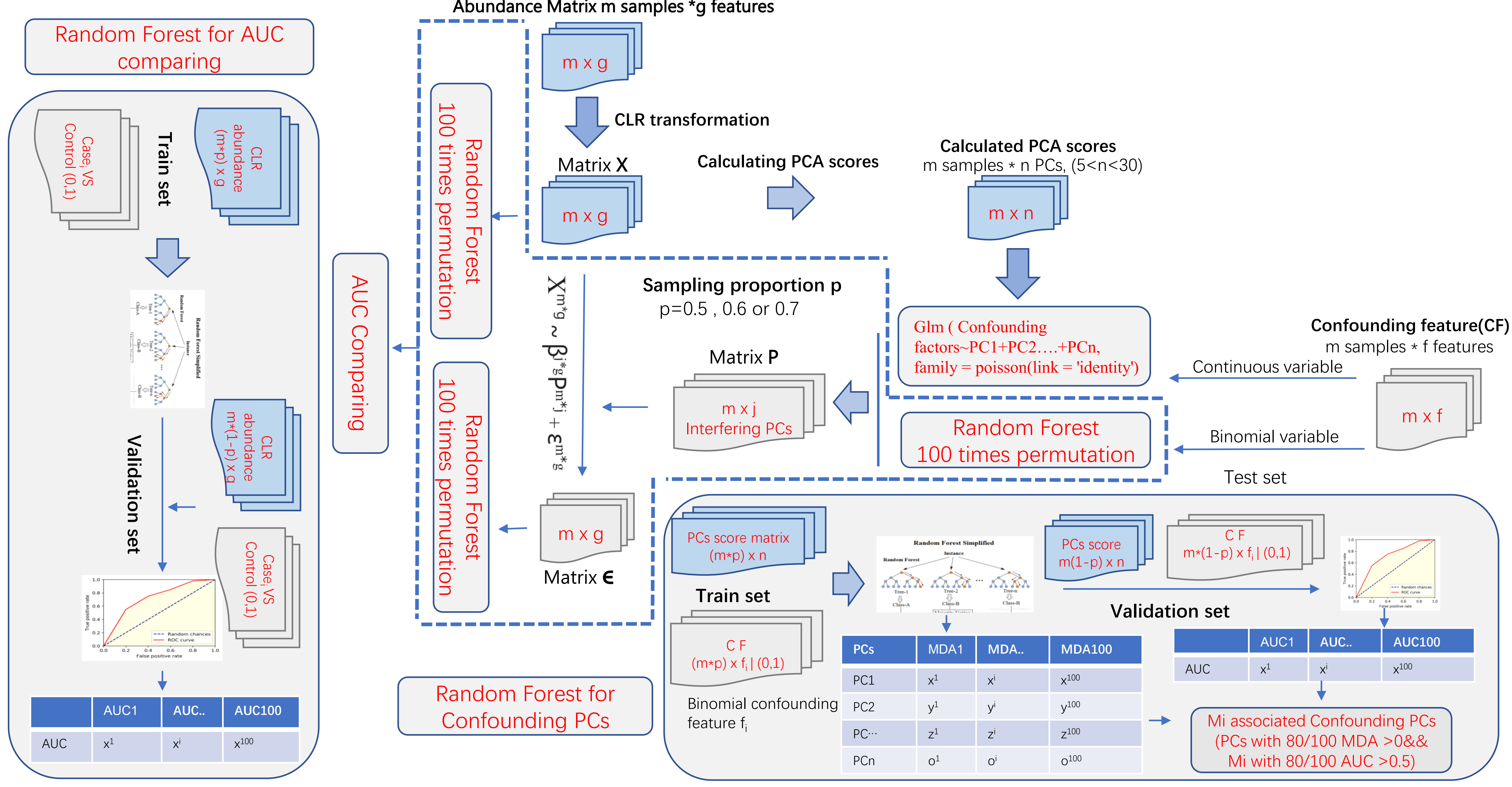
Random Forest for AUC comparing
Case, VS Control (0,1)
Train set
CLR abundance (m*p) x g
CLR abundance m*(1-p) x g
Validation set
Case, VS Control (0,1)
AUC1
AUC..
AUC100
AUC
x^1
x^i
x^{100}
AUC Comparing
Random Forest 100 times permutation
Random Forest 100 times permutation
Abundance Matrix m samples *g features
m x g
CLR transformation
Matrix X
m x g
Calculating PCA scores
Calculated PCA scores
m samples * n PCs, (5<n<30)
m x n
$X^{m*g} \sim \beta^{j*g} P^{m*j} + \varepsilon^{m*g}$
Sampling proportion p
p=0.5 , 0.6 or 0.7
Matrix P
m x j
Interfering PCs
Glm (Confounding factors~PC1+PC2….+PCn, family = poisson(link = 'identity')
Confounding feature(CF)
m samples * f features
Continuous variable
Binomial variable
m x f
Random Forest 100 times permutation
Test set
m x g
Matrix ϵ
PCs score matrix (m*p) x n
Train set
C F (m*p) x f_i | (0,1)
Binomial confounding feature f_i
PCs score m(1-p) x n
C F m*(1-p) x f_i | (0,1)
Validation set
PCs
MDA1
MDA..
MDA100
PC1
PC2
PC…
PCn
x^1 x^i x^{100}
y^1 y^i y^{100}
z^1 z^i z^{100}
o^1 o^i o^{100}
Mi associated Confounding PCs (PCs with 80/100 MDA >0&& Mi with 80/100 AUC >0.5)
Random Forest for Confounding PCs

Figure S3

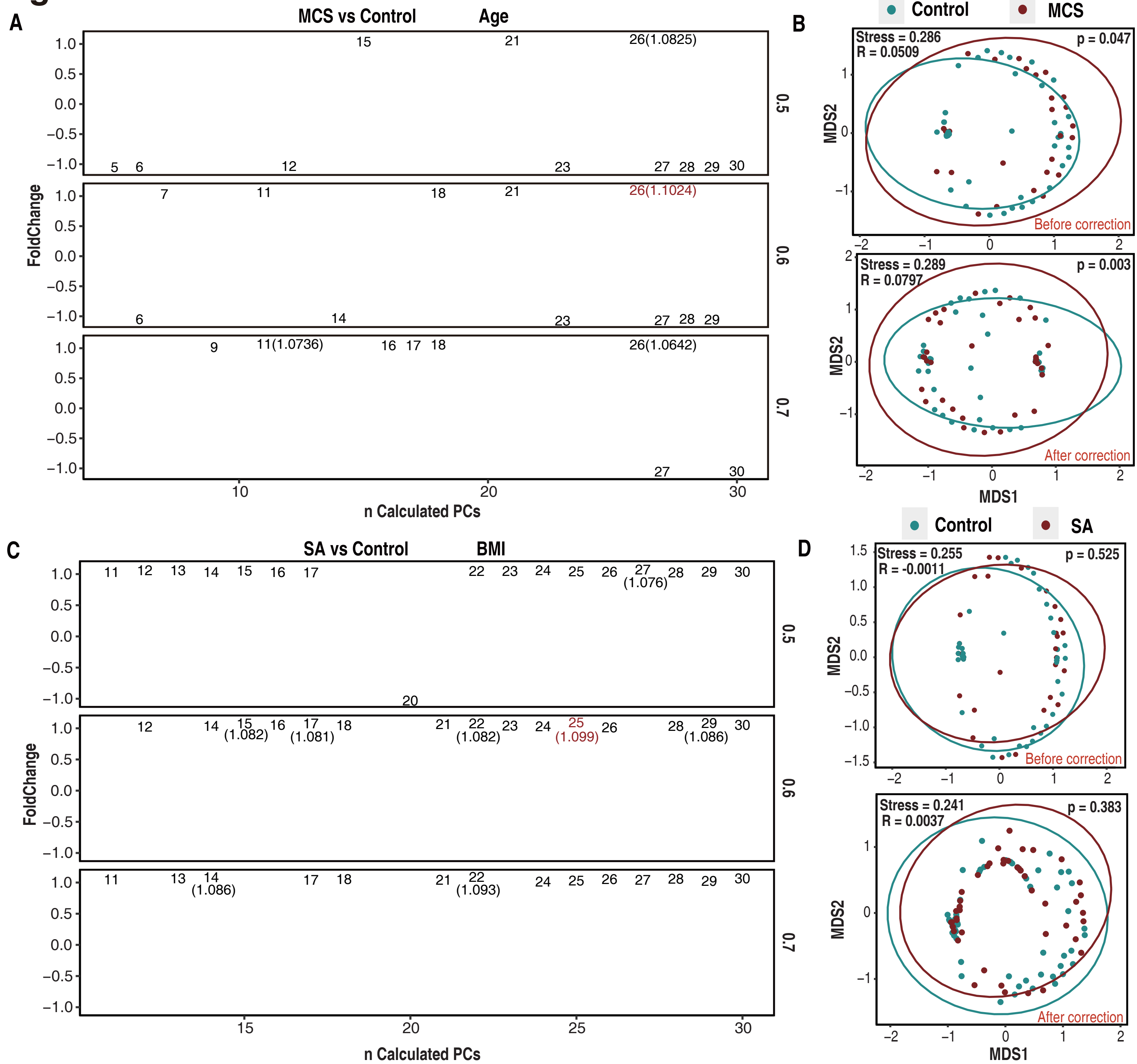

A
MCS vs Control
Age
FoldChange
n Calculated PCs
26(1.0825)
26(1.1024)
11(1.0736)
26(1.0642)
0.5
0.6
0.7
B
Control
MCS
Stress = 0.286
R = 0.0509
p = 0.047
Before correction
Stress = 0.289
R = 0.0797
p = 0.003
After correction
MDS1
MDS2
C
SA vs Control
BMI
FoldChange
n Calculated PCs
27 (1.076)
15 (1.082)
17 (1.081)
22 (1.082)
25 (1.099)
29 (1.086)
14 (1.086)
22 (1.093)
D
Control
SA
Stress = 0.255
R = -0.0011
p = 0.525
Before correction
Stress = 0.241
R = 0.0037
p = 0.383
After correction
MDS1
MDS2

Figure S4

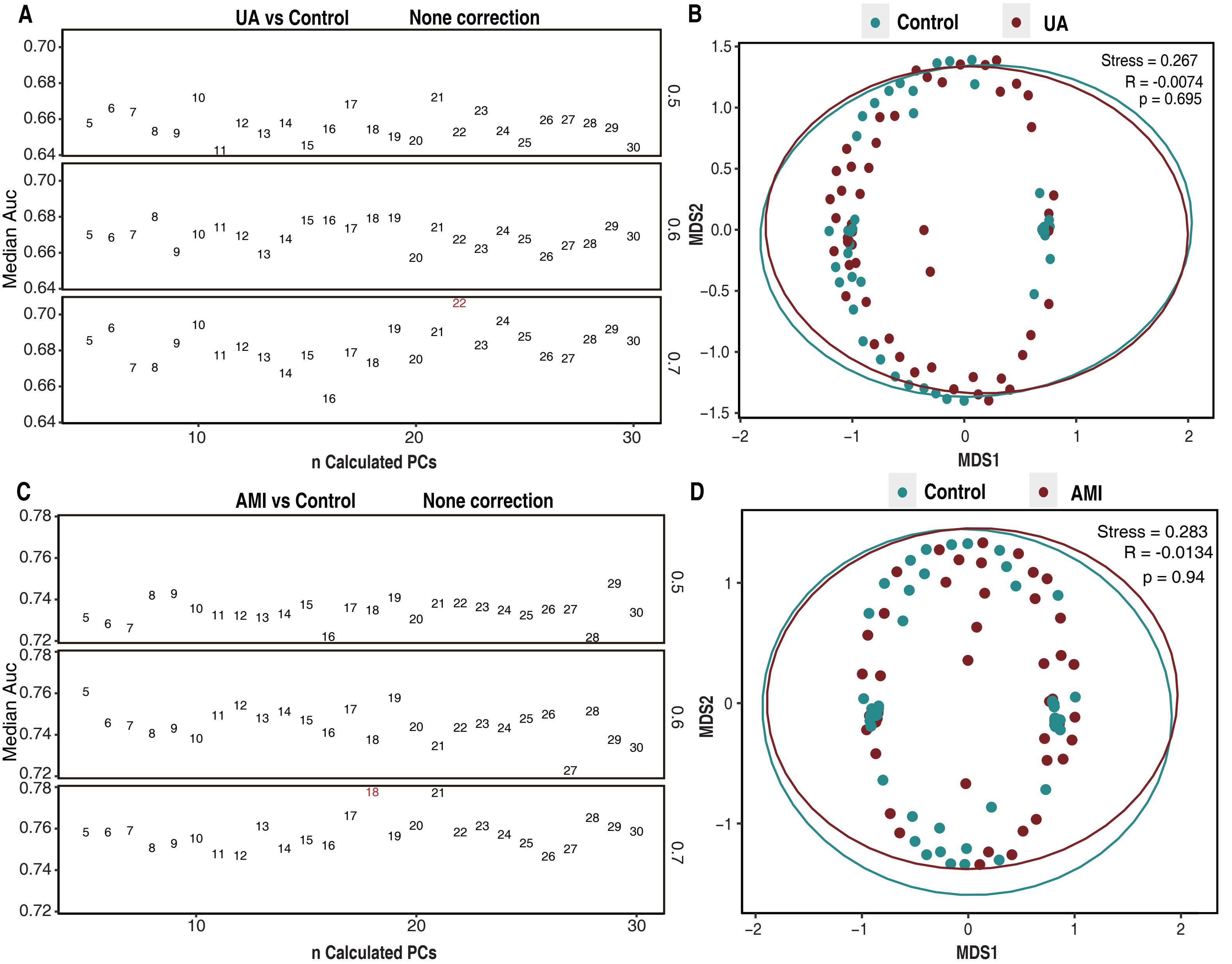

A
UA vs Control
None correction
Median Auc
n Calculated PCs
0.5
0.6
0.7
B
Control
UA
Stress = 0.267
R = -0.0074
p = 0.695
MDS1
MDS2
C
AMI vs Control
None correction
Median Auc
n Calculated PCs
D
Control
AMI
Stress = 0.283
R = -0.0134
p = 0.94
MDS1
MDS2

Figure S5

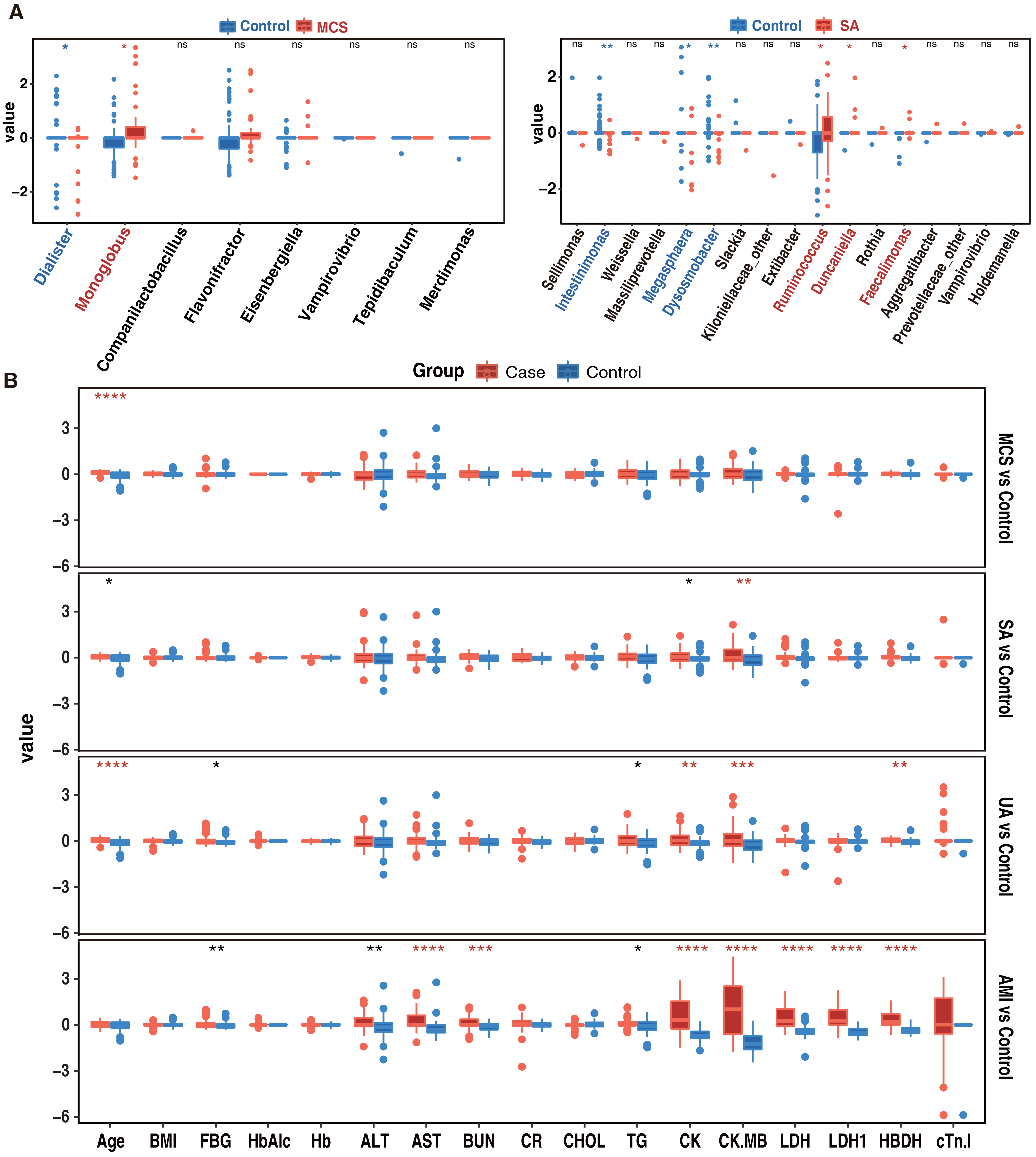
A
Control
MCS
SA
value
Dialister
Monoglobus
Companilactobacillus
Flavonifractor
Eisenbergiella
Vampirovibrio
Tepidibaculum
Merdimonas
Sellimonas
Intestinimonas
Weissella
Massiliprevotella
Megasphaera
Dysosmobacter
Slackia
Kiloniellaceae_other
Extibacter
Ruminococcus
Duncaniella
Rothia
Faecalimonas
Aggregatibacter
Prevotellaceae_other
Vampirovibrio
Holdemanella
B
Group
Case
Control
value
MCS vs Control
SA vs Control
UA vs Control
AMI vs Control
Age
BMI
FBG
HbAlc
Hb
ALT
AST
BUN
CR
CHOL
TG
CK
CK.MB
LDH
LDH1
HBDH
cTn.I

Figure S6

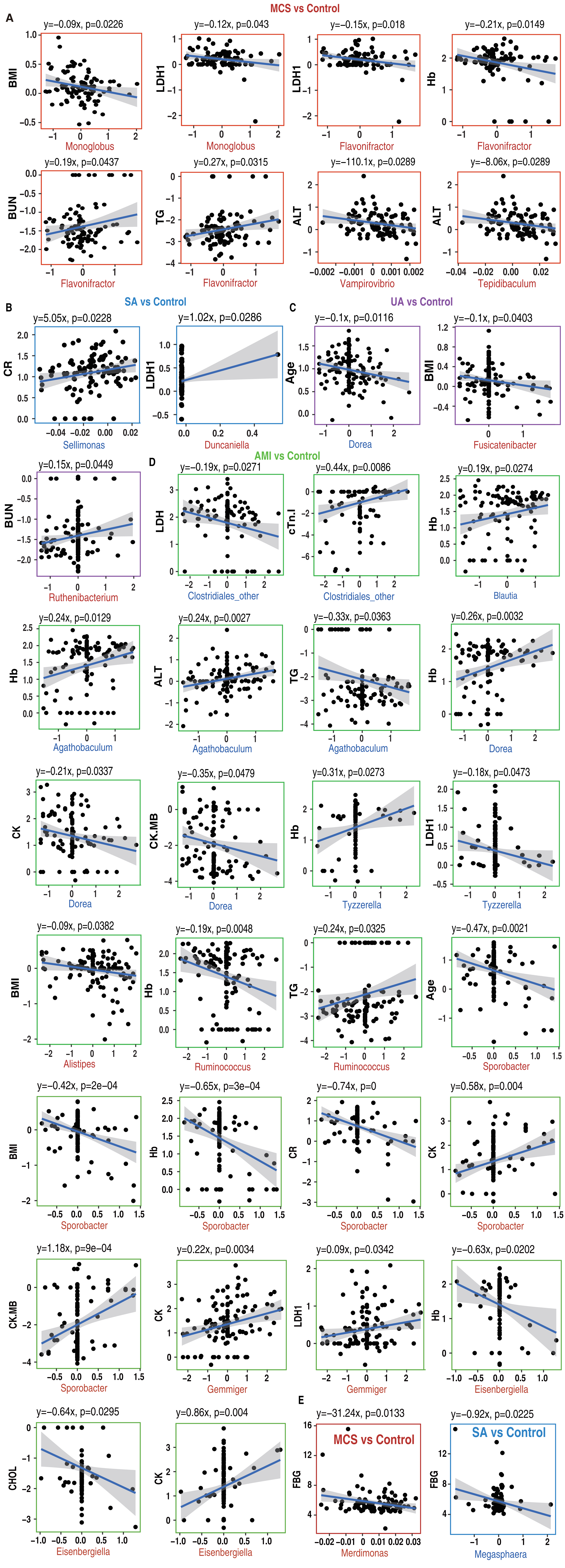

A
MCS vs Control
y=−0.09x, p=0.0226
BMI
Monoglobus
y=−0.12x, p=0.043
LDH1
Monoglobus
y=−0.15x, p=0.018
LDH1
Flavonifractor
y=−0.21x, p=0.0149
Hb
Flavonifractor
y=0.19x, p=0.0437
BUN
Flavonifractor
y=0.27x, p=0.0315
TG
Flavonifractor
y=−110.1x, p=0.0289
ALT
Vampirovibrio
y=−8.06x, p=0.0289
ALT
Tepidibaculum
B
SA vs Control
y=5.05x, p=0.0228
CR
Sellimonas
y=1.02x, p=0.0286
LDH1
Duncaniella
C
UA vs Control
y=−0.1x, p=0.0116
Age
Dorea
y=−0.1x, p=0.0403
BMI
Fusicatenibacter
y=0.15x, p=0.0449
BUN
Ruthenibacterium
D
AMI vs Control
y=−0.19x, p=0.0271
LDH
Clostridiales_other
y=0.44x, p=0.0086
cTn.I
Clostridiales_other
y=0.19x, p=0.0274
Hb
Blautia
y=0.24x, p=0.0129
Hb
Agathobaculum
y=0.24x, p=0.0027
ALT
Agathobaculum
y=−0.33x, p=0.0363
TG
Agathobaculum
y=0.26x, p=0.0032
Hb
Dorea
y=−0.21x, p=0.0337
CK
Dorea
y=−0.35x, p=0.0479
CK.MB
Dorea
y=0.31x, p=0.0273
Hb
Tyzzerella
y=−0.18x, p=0.0473
LDH1
Tyzzerella
y=−0.09x, p=0.0382
BMI
Alistipes
y=−0.19x, p=0.0048
Hb
Ruminococcus
y=0.24x, p=0.0325
TG
Ruminococcus
y=−0.47x, p=0.0021
Age
Sporobacter
y=−0.42x, p=2e−04
BMI
Sporobacter
y=−0.65x, p=3e−04
Hb
Sporobacter
y=−0.74x, p=0
CR
Sporobacter
y=0.58x, p=0.004
CK
Sporobacter
y=1.18x, p=9e−04
CK.MB
Sporobacter
y=0.22x, p=0.0034
CK
Gemmiger
y=0.09x, p=0.0342
LDH1
Gemmiger
y=−0.63x, p=0.0202
Hb
Eisenbergiella
y=−0.64x, p=0.0295
CHOL
Eisenbergiella
y=0.86x, p=0.004
CK
Eisenbergiella
E
y=−31.24x, p=0.0133
MCS vs Control
FBG
Merdimonas
y=−0.92x, p=0.0225
SA vs Control
FBG
Megasphaera

SUPPLEMENTAL MATERIAL

Supplementary Table S1-S12

Supplementary figure 1. Rarecurve of richness when sampled 1000 reads per step per sample. Sampling at 10000 even depth was found sufficient for most OTUs.

Supplementary figure 2. Flowchart for interfering factors identification, correction and estimation of correction performance according to AUC in classification of Case/Control samples after correction.

Supplementary figure 3. (A) Foldchange at each n-p parameter combination where AUC was significantly increased after correction of Age in MCS vs Control. The highest AUC elevation according to median AUC was marked in red. (B) NMDS and ANOSIM analysis were performed with genus CLR abundance in MCS vs Control before and after Age correction with n=26, p=0.6. (C) Foldchange at each n-p parameter combination where AUC was significantly increased after correction of BMI in SA vs Control. The highest AUC elevation according to median AUC was marked in red. (D) NMDS and ANOSIM analysis were performed with genus CLR abundance in SA vs Control before and after BMI correction with n=25, p=0.6.

Supplementary figure 4. (A) As there was no noise associated interference was identified in UA vs Control. With 2 random runs with none factors corrected with each of n-p parameter combinations, the highest AUC according to median AUC in the second random run was marked in red. (B) NMDS and ANOSIM analysis were performed with genus CLR abundance in UA vs Control with 2 random runs (C) Similar with that in UA vs Control, no noise associated interference was identified in AMI vs Control. Thus, with 2 random runs with none factors corrected with each of n-p parameter combinations, the highest AUCs according to median AUC in the second random run was marked in red. (D) NMDS and ANOSIM analysis were performed with genus CLR abundance in AMI vs Control. It was note that, 2 random runs were performed with random selected samples with different n-p parameter combination.

Supplementary figure 5. (A) Comparison of indicator genus between case and control samples with abundance before noise correction in MCS vs Control and SA vs Control groups. *: $0.01<p<0.05$, **: $0.001<p<0.01$, ***: $0.0001<p<0.001$, ****: $p<0.0001$.

Wilcoxon Test (B) Difference of clinical indexes between case and control samples in MCS vs Control, SA vs Control, UA vs Control and AMI vs Control groups. Considering the huge value magnitude for different clinical indexes, Values of same clinical index were CLR normalized for comparison between case and control samples in 4 groups. *: $0.01<p<0.05$, **: $0.001<p<0.01$, ***: $0.0001<p<0.001$, ****: $p<0.0001$. Wilcoxon Test. Comparison with p value passing Benjamini-Hochberg adjustment were marked in red stars.

Supplementary figure 6. (A) Correlations between indicator genus and clinical indexes in MCS vs Control group. (B) Correlations between indicator genus and clinical indexes in SA vs Control group. (C) Correlations between indicator genus and clinical indexes in UA vs Control group. (D)Correlations between indicator genus and clinical indexes in AMI vs Control group. (E) Correlation between FBG and indicator genus in MCS vs Control and SA vs Control. Plot box were colored according to groups, correlations in MCS vs Control group were showed in red plot box; correlations in SA vs Control group were showed in blue plot box; correlations in UA vs Control group were showed in purple plot box; correlations in AMI vs Control group were showed in green plot box. Genus with red x-axis labels suggested the correlations were disease indicator associated while genus with blue axis labels suggested the correlations were control indicator associated.
