# Supplementary material for: Diversified Shifts in the Cross Talk between Members of the Gut Microbiota and Development of Coronary Artery Diseases
Source: Microbiol Spectr. 2022 Oct 27;10(6):e02804-22. doi: 10.1128/spectrum.02804-22 (PMC9769841; doi:10.1128/spectrum.02804-22)
Supplement: Reviewer comments [file reviewer-comments.pdf]

AMERICAN SOCIETY FOR MICROBIOLOGY | Microbiology Spectrum

Diversified shifts in the crosstalk between gut microbiota and development of coronary artery diseases

Tao Zhang, Haiqing Ren, Zhihui Du, Tong Zou, Xuefeng Guang, Yanan Zhang, Yuqing Tian, Lei Zhu, Jiangkun Yu, Xue Yu, Zhigang Zhang, and Hailong Dai

Corresponding Author(s): Zhigang Zhang, Yunnan University

Review Timeline:

Submission Date:	July 21, 2022
Editorial Decision:	August 14, 2022
Revision Received:	September 8, 2022
Accepted:	September 30, 2022

Editor: Wei-Hua Chen

Reviewer(s): Disclosure of reviewer identity is with reference to reviewer comments included in decision letter(s). The following individuals involved in review of your submission have agreed to reveal their identity: Chaoran Dong (Reviewer #1); Evgenii I. Olekhnovich (Reviewer #2)

Transaction Report:

(Note: With the exception of the correction of typographical or spelling errors that could be a source of ambiguity, letters and reports are not edited. The original formatting of letters and referee reports may not be reflected in this compilation.)

DOI: https://doi.org/10.1128/spectrum.02804-22

August 14, 2022

Prof. Zhigang Zhang
Yunnan University
No. 2, Cuihu North Road, Wuhua District
Kunming, Yunnan Province 650106
China

Re: Spectrum02804-22 (Diversified shifts in the crosstalk between gut microbiota and development of coronary artery diseases)

Dear Prof. Zhigang Zhang:

Thank you for submitting your manuscript to Microbiology Spectrum. Your paper has been evaluated by two external experts. Although they found your research interesting, they have also raised substantial concerns. I thus would like to invite you to submit a revised version of your paper to properly and sufficiently address their concerns.

When submitting the revised version of your paper, please provide (1) point-by-point responses to the issues raised by the reviewers as file type "Response to Reviewers," not in your cover letter, and (2) a PDF file that indicates the changes from the original submission (by highlighting or underlining the changes) as file type "Marked Up Manuscript - For Review Only". Please use this link to submit your revised manuscript - we strongly recommend that you submit your paper within the next 60 days or reach out to me. Detailed instructions on submitting your revised paper are below.

Link Not Available

Below you will find instructions from the Microbiology Spectrum editorial office and comments generated during the review.

ASM policy requires that data be available to the public upon online posting of the article, so please verify all links to sequence records, if present, and make sure that each number retrieves the full record of the data. If a new accession number is not linked or a link is broken, provide production staff with the correct URL for the record. If the accession numbers for new data are not publicly accessible before the expected online posting of the article, publication of your article may be delayed; please contact the ASM production staff immediately with the expected release date.

The ASM Journals program strives for constant improvement in our submission and publication process. Please tell us how we can improve your experience by taking this quick Author Survey.

Sincerely,

Wei-Hua Chen

Editor, Microbiology Spectrum

Journals Department
American Society for Microbiology
1752 N St., NW
Washington, DC 20036
E-mail: spectrum@asmusa.org

Reviewer comments:

Reviewer #1 (Comments for the Author):

The current study by Zhang et al. explored and identified the disease or noise roles for several confounding factors in regulating gut microbial in development of different CAD diseases and identical indicator microbes after noise correction might have important potentials for diagnosis or therapy of cardiovascular disorders. The study was well designed and the data was interesting.

There are few specific points as below:
1) The clinical cohort grouping was interesting. It would be more meaningful to further discuss the differences in gut microbiota

among different groups, and to establish disease diagnostic models.
2) Gut microbiota played the role in CAD mainly through metabolites. If the author could supplement the corresponding metabolomics data, the study would be more comprehensive.
3) In the discussion section, author need to further discuss the role and significance of gut microbiota in coronary heart disease based on previous research.
4) The authors identified and presented disease or control indicators after noise correction and I'm very interested in the disease or control indicator before noise correction. Maybe add the bacterial indicators result without factors correction and have a comparison with that after noise correction would make it better in work.
5) Some description need to be clearer, for example 'Though community types gathered samples with similar gut microbiota compositions, ..., to disease development or just background noise was inconclusive.' from line 94 to 98, and "estimated the disease or noise associated ..... with a PCA and Random Forest based method" from line 107 to 109.
6) From line 119 to 121, the sentence "And with 97% sequence identity threshold, a total of 3644 OTUs with 167{plus minus}64 OTUs per sample were finally obtained" told the downstream analysis were performed based on OTUs while in the method section, AVS were acquired. Please conform the right method.
7) The capitalization of some words was not identical over all manuscript, for example "Case/Control" in line 184, "case/control" in line 194, "case/control" in line 291 , "case/control" in line 194, 292, 394 ... 835, and "Case/Control" in line 861. The authors should make it identical.
8) Many sentences should be written in a much more concise way. From line 134 to 145, 'In identifying interfering factors....... tend to increase (Figure 1B, Table S4)', From line 252 to line 256, 'In MCS vs Control......parameter combinations', and from line 309 to line 320,' In SA vs Control ..... identified SA indicators'. A Language polishing would be recommended.

Reviewer #2 (Comments for the Author):

Zhang et al. presented an interesting paper titled "Diversified shifts in the crosstalk between gut microbiota and development of coronary artery diseases". Authors revealed the disease or noise roles for several confounding factors in regulating gut microbial in development of different CAD diseases. In addition, markers microbes linked to CAD after noise correction were identified. I found this work potentially useful both in the microbiome and medical research fields. However, I would advise authors to consider some remarks presented further that may be useful in improving the manuscript.

Generally manuscript is written well, however it is difficult in some parts to understand the connection between the figures and the text. I think that some figures require re-building for a better understanding of the text.

Remarks:
1) I did not find detailed information on patients with type 2 diabetes. Was the patient's diabetes compensated? To what extent might the glycated hemoglobin index affect the assessment of the significance of type 2 diabetes as a confounding factor in your sample of patients? If you have these clinical data, it would be interesting to add this index to the analysis of clinical indicators.

2) Please add the X axis title to Figure 4. In addition, it is difficult to understand links between the text and Figure 4. For example, Figure 4A consists of 3 sub-figures, it may be worth assigning a letter index to each.

3) Figure 5 also requires a redesign as it is very hard to read and understand.

4) line 353-371: Was it multiple hypothesis testing? If so, was multiple comparison adjustment applied in the clinical index analysis in addition to the Wilcoxon test?

5) line 80: Aerobacte → Aerobacter

6) line 662: DATA2 → DADA2

Staff Comments:

Preparing Revision Guidelines
To submit your modified manuscript, log onto the eJP submission site at https://spectrum.msubmit.net/cgi-bin/main.plex. Go to Author Tasks and click the appropriate manuscript title to begin the revision process. The information that you entered when you first submitted the paper will be displayed. Please update the information as necessary. Here are a few examples of required updates that authors must address:

• Point-by-point responses to the issues raised by the reviewers in a file named "Response to Reviewers," NOT IN YOUR COVER LETTER.
• Upload a compare copy of the manuscript (without figures) as a "Marked-Up Manuscript" file.

• Each figure must be uploaded as a separate file, and any multipanel figures must be assembled into one file.
• Manuscript: A .DOC version of the revised manuscript
• Figures: Editable, high-resolution, individual figure files are required at revision, TIFF or EPS files are preferred

For complete guidelines on revision requirements, please see the journal Submission and Review Process requirements at https://journals.asm.org/journal/Spectrum/submission-review-process. **Submissions of a paper that does not conform to Microbiology Spectrum guidelines will delay acceptance of your manuscript.** "

Please return the manuscript within 60 days; if you cannot complete the modification within this time period, please contact me. If you do not wish to modify the manuscript and prefer to submit it to another journal, please notify me of your decision immediately so that the manuscript may be formally withdrawn from consideration by Microbiology Spectrum.

If your manuscript is accepted for publication, you will be contacted separately about payment when the proofs are issued; please follow the instructions in that e-mail. Arrangements for payment must be made before your article is published. For a complete list of **Publication Fees**, including supplemental material costs, please visit ourwebsite.

Corresponding authors may join or renew ASM membership to obtain discounts on publication fees. Need to upgrade your membership level? Please contact Customer Service at Service@asmusa.org.

Thank you for submitting your paper to Microbiology Spectrum.

The current study by Zhang et al. explored and identified the disease or noise roles for several confounding factors in regulating gut microbial in development of different CAD diseases and identical indicator microbes after noise correction might have important potentials for diagnosis or therapy of cardiovascular disorders. The study was well designed and the data was interesting.

There are few specific points as below:

1) The clinical cohort grouping was interesting. It would be more meaningful to further discuss the differences in gut microbiota among different groups, and to establish disease diagnostic models.
2) Gut microbiota played the role in CAD mainly through metabolites. If the author could supplement the corresponding metabolomics data, the study would be more comprehensive.
3) In the discussion section, author need to further discuss the role and significance of gut microbiota in coronary heart disease based on previous research.
4) The authors identified and presented disease or control indicators after noise correction and I'm very interested in the disease or control indicator before noise correction. Maybe add the bacterial indicators result without factors correction and have a comparison with that after noise correction would make it better in work.
5) Some description need to be clearer, for example 'Though community types gathered samples with similar gut microbiota compositions, ……, to disease development or just background noise was inconclusive.' from line 94 to 98, and "estimated the disease or noise associated ….. with a PCA and Random Forest based method" from line 107 to 109.
6) From line 119 to 121, the sentence "And with 97% sequence identity threshold, a total of 3644 OTUs with 167±64 OTUs per sample were finally obtained" told the downstream analysis were performed based on OTUs while in the method section, AVS were acquired. Please conform the right method.
7) The capitalization of some words was not identical over all manuscript, for

example “Case/Control” in line 184, “case/control” in line 194, “case/control” in line 291 , “case/control” in line 194, 292, 394 … 835, and “Case/Control” in line 861. The authors should make it identical.

8) Many sentences should be written in a much more concise way. From line 134 to 145, ‘In identifying interfering factors……. tend to increase (Figure 1B, Table S4)’, From line 252 to line 256, ‘In MCS vs Control……parameter combinations’, and from line 309 to line 320,’ In SA vs Control ….. identified SA indicators’. A Language polishing would be recommended.

Reviewer #1 (Comments for the Author):

The current study by Zhang et al. explored and identified the disease or noise roles for several confounding factors in regulating gut microbial in development of different CAD diseases and identical indicator microbes after noise correction might have important potentials for diagnosis or therapy of cardiovascular disorders. The study was well designed and the data was interesting.

There are few specific points as below:

1) The clinical cohort grouping was interesting. It would be more meaningful to further discuss the differences in gut microbiota among different groups, and to establish disease diagnostic models.
Response: As reviewer recommended, we established disease diagnostic models for each group with gut microbial indicators based on corrected abundance using the random forest algorithm. The AUC values for model accuracy were 0.829, 0.762, 0.679, and 0.827 respectively for MCS vs Control, SA vs Control, UA vs Control and AMI vs Control (**See new added Figure 5**). Besides solely relying on microbial indicators, diagnostic models solely based on clinical indexes as well as that applying both microbial indicators and clinical indexes were also established. Distinct predicting performances in our results were showed in different groups. For MCS vs Control group, the highest AUC value is 0.829 solely returned by microbial indicators. Similarly, the highest AUC value 0.762 is also returned by microbial indicators alone for SA vs Control group. These results indicate the diagnostic potential for gut microbes in early-stage CAD diseases. For UA vs Control group, the consistent highest AUC value 0.75 is obtained according to the clinical indexes or the combination of clinical indexes which was higher than microbial indicators. This finding indicated that microbial indicator cannot improve the UA diagnosis which was based on clinical indexes. In contrast, for AMI vs Control group, the AUC values were 0.843 (clinical indexes and microbial indicators), 0.807 (microbial indicators), and 0.721 (clinical indexes), respectively. This result suggests that microbial indicator can return better performance than clinical indexes and increase the diagnosis resolution of AMI based on clinical indexes. We added new results (**Lines 392-407**), related discussions (**Lines 572-586**) and figure legend (**Lines 888-897**) for newly added **Figure 5** (see below) in the revised text.

Figure 5

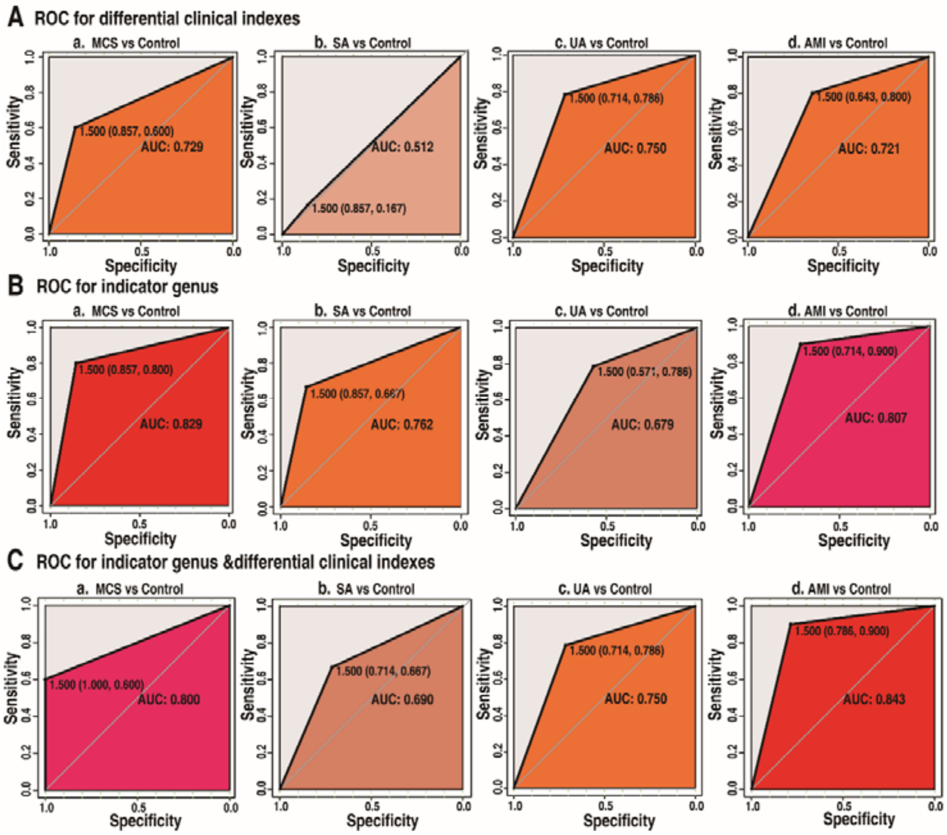

2) Gut microbiota played the role in CAD mainly through metabolites. If the author could supplement the corresponding metabolomics data, the study would be more comprehensive.

Response: This is a good suggestion but here we are sorry to say that we did not collect the serum or urine samples when performing this project. Only fresh fecal samples were collected for performing 16S RNA-based amplicon sequencing and preserved in the tube with the DNA Protection Solution at 80 ℃, which are also unsuitable for further metabolic analysis. In our upcoming project, we will perform multiple-omics analyses of serum, urinal, and fecal samples from CAD patients according to referee suggestions for obtaining high-confident markers for the diagnosis or treatment of CAD diseases.

3) In the discussion section, author need to further discuss the role and significance of gut microbiota in coronary heart disease based on previous research.

Response: we added discussions (**Lines 566-568** and **Lines 617-618**) about the role of microbes according to referee suggestions.

4) The authors identified and presented disease or control indicators after noise correction and I'm very interested in the disease or control indicator before noise correction. Maybe add the bacterial indicators result without factors correction and

have a comparison with that after noise correction would make it better in work.

Response: As reviewer recommended, for noise correction were just performed in MCS vs Control and SA vs Control groups, with indicator genus identified in the 2 groups, comparison between case and control samples were performed with un-corrected values. As showed in newly added Figure S5A, there was difference in significance in MCS vs Control, but few in SA vs Control, suggesting the varied noise influence in different groups. Related results have been added in the revised text (**Lines 351-356**).

5) Some description need to be clearer, for example 'Though community types gathered samples with similar gut microbiota compositions, ..., to disease development or just background noise was inconclusive.' from line 94 to 98, and "estimated the disease or noise associated ..... with a PCA and Random Forest based method" from line 107 to 109.

Response: As reviewer recommended, we revised these sentences for a better reading and understanding. See **Lines 101-106** and **Lines 114-119**.

6) From line 119 to 121, the sentence "And with 97% sequence identity threshold, a total of 3644 OTUs with 167±64 OTUs per sample were finally obtained" told the downstream analysis were performed based on OTUs while in the method section, AVS were acquired. Please conform the right method.

Response: Sorry for this error and the method should be DADA2 but not TADA2. This error has been corrected (**Line 689**).

7) The capitalization of some words was not identical over all manuscript, for example

"Case/Control" in line 184, "case/control" in line 194, "case/control" in line 291 , "case/control" in line 194, 292, 394 ... 835, and "Case/Control" in line 861. The authors should make it identical.

Response: Sorry for that negligence and they were corrected. (**Line191, Line201, Line292, Line293, Line352, Line 424, Line736, Line850, Line851, Line856, Line878, Line925**)

8) Many sentences should be written in a much more concise way. From line 134 to 145, 'In identifying interfering factors....... tend to increase (Figure 1B, Table S4)', From line 252 to line 256, 'In MCS vs Control......parameter combinations', and from line 309 to line 320,' In SA vs Control ..... identified SA indicators'. A Language polishing would be recommended.

Response: As reviewer recommended, we improved the writing of these sentences (**Lines 142-147, Lines 254-261, and Lines 306-324**) for better reading and understanding.

Reviewer #2 (Comments for the Author):

Zhang et al. presented an interesting paper titled "Diversified shifts in the crosstalk between gut microbiota and development of coronary artery diseases". Authors revealed the disease or noise roles for several confounding factors in regulating gut microbial in development of different CAD diseases. In addition, markers microbes linked to CAD after noise correction were identified. I found this work potentially useful both in the microbiome and medical research fields. However, I would advise authors to consider some remarks presented further that may be useful in improving the manuscript.

Generally manuscript is written well, however it is difficult in some parts to understand the connection between the figures and the text. I think that some figures require re-building for a better understanding of the text.

Remarks:

1) I did not find detailed information on patients with type 2 diabetes. Was the patient's diabetes compensated? To what extent might the glycated hemoglobin index affect the assessment of the significance of type 2 diabetes as a confounding factor in your sample of patients? If you have these clinical data, it would be interesting to add this index to the analysis of clinical indicators.

Response: We greatly appreciated the suggestions by the referee. Accordingly, we added the FBG (Fasting Blood Glucose) and HbAlc (Glycosylated Hemoglobin) information of CAD patients in Table S1. We found the FBG showed significant difference (p value < 0.05, BH adjusted $p > 0.05$, Wilcoxon Test) both in UA vs Control group and in AMI vs Control group while no significant difference was observed both in MCS vs Control group and SA vs Control group (see new added Figure S5B). Due to the lack of HbAlc measurements from most of patients, we found no significant difference of HbAlc between case and control samples in each of 4 groups, which was needed to be further confirmed in the upcoming project. For correlation analysis, based on normalized the FBG values, we found no significant associations between FBG and indicator genus in 4 groups. But the FBG without normalization showed significantly negative association with MCS indicator *Merdimonas* in MCS vs Control and control indicator (vs SA) *Megasphaera* in SA vs Control (Figure S6D). Due to the FBG closely related to the risk and severity of coronary heart disease, it's not surprised to find the negative connection with FBG of control indicator (vs SA) *Megasphaera*. However, it's interesting to found negative linkage between FBG and MCS indicator. As discussed earlier in BMI correlation analysis that MCS indicators or even MCS gut microbes may likely play a favorable role in response to transition of MCS status from health state. The negative association between FBG and the MCS indicator *Merdimonas* further supported a possibility about the beneficial role of gut microbes in MCS patients.

As for significant associations between microbe indicators and FBG were just found in MCS vs Control as well as SA vs Control groups in which FBG showed no significant Case/Control differences rather than in groups, such as UA vs Control and AMI vs Control, that FBG showed significant Case/Control differences, maybe host affection played more roles. We add results in **Lines 360-361, Line 381 and Lines 415-420, related discussion in Lines 617-626**)

2) Please add the X axis title to Figure 4. In addition, it is difficult to understand links between the text and Figure 4. For example, Figure 4A consists of 3 sub-figures, it may be worth assigning a letter index to each.

Response: Thanks for this suggestion. In the revised Figure 4, we assigned a letter index combined with X-axis title to each sub-figure. To better understand the Figure 4, we added more detailed descriptions in the revised text (**Lines 304-305, Lines 320-322**) and much more information in the figure legend. (**Lines 874-883**)

3) Figure 5 also requires a redesign as it is very hard to read and understand.

Response: Thanks for this comment. As Reviewer recommended, we revised Figure 5 and labeled as Figure 6 to be more briefly read and easily understood. (**Lines 415-420, Lines 424-433, Lines 899-917**)

4) line 353-371: Was it multiple hypothesis testing? If so, was multiple comparison adjustment applied in the clinical index analysis in addition to the Wilcoxon test?

Response: Yes, we performed a multiple hypothesis testing in lines 353-371. The Wilcoxon test was applied in comparison between case and control group without multiple comparison adjustment. As reviewer mentioned it, we further adjusted these p values for significance in clinical indicator analysis. As showed in Figure S5B, raw significant *p* values lower that 0.05 were marked with black stars and adjusted *p* values lower than 0.05 with the Benjamini-Hochberg method were colored by red stars. See **Lines 367-384** and **Lines 953-955** in the revised text.

5) line 80: Aerobacte → Aerobacter

Response: Sorry for this mistake. It was corrected in **Line 85** in the revised text.

6) line 662: DATA2 → DADA2

Response: Sorry for this mistake. It was corrected in the revised text (**Line 689**).

September 30, 2022

Prof. Zhigang Zhang
Yunnan University
No. 2, Cuihu North Road, Wuhua District
Kunming, Yunnan Province 650106
China

Re: Spectrum02804-22R1 (Diversified shifts in the crosstalk between gut microbiota and development of coronary artery diseases)

Dear Prof. Zhigang Zhang:

Congratulations. Based on the comments of the two external reviewers, I am glad to accept your manuscript for publication in Microbiology Spectrum. I am forwarding it to the ASM Journals Department for publication. You will be notified when your proofs are ready to be viewed.

I would also like to take the chance to thank the two reviewers for their time and effort to improve the submission.

The ASM Journals program strives for constant improvement in our submission and publication process. Please tell us how we can improve your experience by taking this quick Author Survey.

As an open-access publication, Spectrum receives no financial support from paid subscriptions and depends on authors' prompt payment of publication fees as soon as their articles are accepted. You will be contacted separately about payment when the proofs are issued; please follow the instructions in that e-mail. Arrangements for payment must be made before your article is published. For a complete list of **Publication Fees**, including supplemental material costs, please visit ourwebsite.

ASM policy requires that data be available to the public upon online posting of the article, so please verify all links to sequence records, if present, and make sure that each number retrieves the full record of the data. If a new accession number is not linked or a link is broken, provide production staff with the correct URL for the record. If the accession numbers for new data are not publicly accessible before the expected online posting of the article, publication of your article may be delayed; please contact the ASM production staff immediately with the expected release date.

Corresponding authors may join or renew ASM membership to obtain discounts on publication fees. Need to upgrade your membership level? Please contact Customer Service at Service@asmusa.org.

Thank you for submitting your paper to Spectrum.

Sincerely,

Wei-Hua Chen
Editor, Microbiology Spectrum

Journals Department
American Society for Microbiology
1752 N St., NW
Washington, DC 20036
E-mail: spectrum@asmusa.org

Supplemental Tables: Accept
Supplemental Material: Accept
